# Exploring the Gut and Oral Microbiomes in Psychoactive Substance Use: A Scoping Review of Clinical Studies

**DOI:** 10.1111/jnc.70165

**Published:** 2025-07-25

**Authors:** Artūras Barkus, Vaida Baltrūnienė, Lina Barkienė, Justė Baušienė, Tomas Baltrūnas, Marius Brazys, Kornelija Rauduvytė, Paulina Kazlauskaitė, Augustinas Baušys

**Affiliations:** ^1^ Department of Pathology and Forensic Medicine, Institute of Biomedical Sciences, Faculty of Medicine Vilnius University Vilnius Lithuania; ^2^ Laboratory of Experimental Surgery and Oncology, Translational Health Research Institute, Faculty of Medicine Vilnius University Vilnius Lithuania

**Keywords:** alcohol, cannabis, gut, microbiome, opioids, oral, stimulants, substance use

## Abstract

Substance use disorders (SUDs) constitute a significant global health challenge, and emerging evidence suggests that the gut and oral microbiomes may play significant roles in addiction pathophysiology, yet the human clinical literature remains fragmented. This scoping review systematically synthesizes evidence from 75 clinical studies investigating alterations in gut and oral microbiomes associated with alcohol, stimulant, cannabis, and opioid use. Across studies, beta‐diversity analyses frequently reveal clear differences between substance users and controls, indicating distinct community structures. Findings on alpha diversity and specific taxonomic shifts vary by substance. Commonly observed changes included declines in beneficial short‐chain fatty acid‐producing taxa, alongside expansions of opportunistic or proinflammatory microorganisms. However, substantial methodological heterogeneity, including variations in study design, population characteristics, and analytical methods, complicates direct comparisons and definitive conclusions. Limited longitudinal evidence indicates partial microbiome recovery after extended abstinence, although full restoration remains uncertain. Further longitudinal research with standardized methods is needed to clarify these findings and inform potential microbiome‐targeted therapies for SUDs.

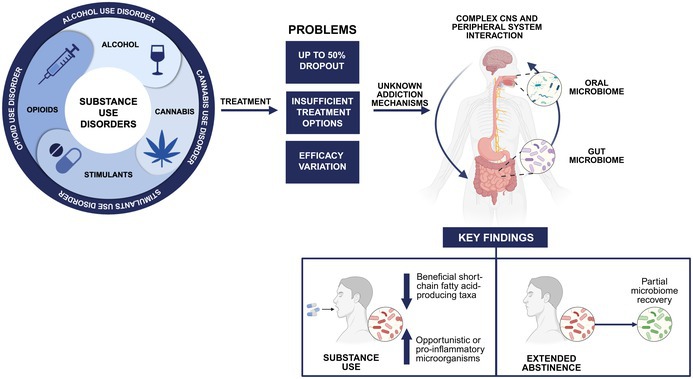

## Graphical abstract

Substance use disorders are linked to high treatment dropout rates and limited therapeutic options. Clinical studies show that substance use disrupts gut and oral microbiomes, decreasing beneficial bacteria and increasing proinflammatory and opportunistic microbes. While limited longitudinal evidence suggests partial microbiome recovery after extended abstinence, full restoration remains uncertain, highlighting the need for standardized methodologies and multiomics approaches to elucidate recovery dynamics and therapeutic potential.

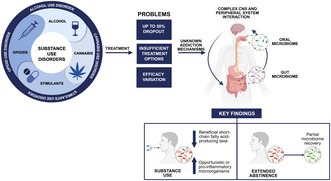

**Abbreviations**
AB: abstinent
ACE: abundance‐based coverage estimator
ACS: American Cancer Society
AD: alcohol dependence
ADS: alcohol dependence syndrome
Ag: opioid agonist
AgAt: opioid agonist and antagonist
Allo‐HCT: allogeneic hematopoietic cell transplantation
ASV: amplicon sequence variant
At: opioid antagonist
AUD: alcohol use disorder
AUDIT: alcohol use disorders identification test
AUDIT‐C: short version of AUDIT
BDs: binge drinkers
Bray–Curtis: Bray–Curtis dissimilarity
CD: current drinkers
CDet: compulsory detention patients
Chao1: Chao1 richness estimator
CNS: central nervous system
CPS II: Cancer Prevention Study II
CRC: colorectal cancer
CUD: cannabis use disorder
CUDIT: cannabis use disorder identification test
DSM‐5: diagnostic and statistical manual of mental disorders, fifth edition
DSM‐IV: diagnostic and statistical manual of mental disorders, fourth edition
DU: drug users
Faith's PD: faith's phylogenetic diversity
FD: former drinkers
FMT: fecal microbiota transplantation
GWAS: genome‐wide association study
HC: healthy controls
HD: heavy drinkers
HDC: heavy drinking controls
Heip: Heip's evenness index
HE Op: patients with hepatic encephalopathy on opioids
HIV: human immunodeficiency virus
ICD‐10: international classification of diseases, tenth revision
ICU: intensive care unit
ITS1: internal transcribed spacer 1
Jaccard: Jaccard distance
Jensen–Shannon: Jensen–Shannon distance
LD: light drinkers
LefSe: Linear discriminant analysis Effect Size
LHD: less heavy drinkers
MA: methamphetamine
MA‐GS: methamphetamine—good sleep group
Margalef: Margalef richness index
MD: moderate drinkers
MDMA: 3,4‐methylenedioxymethamphetamine
MELD: model for end‐stage liver disease
MMT: methadone maintenance therapy
MP: MMT patients
MSM: men who have sex with men
MUD: methamphetamine use disorder
N: neither opioid agonist nor antagonist
NA: not applicable/not available
NAFLD: nonalcoholic fatty liver disease
NCI: National Cancer Institute
ND: never drinkers
NetoVIR: NetoVIR viral metagenomics pipeline
NIAAA: National Institute on Alcohol Abuse and Alcoholism
NIH: National Institutes of Health
Non‐HE Op: patients without hepatic encephalopathy on opioids
NOS: Newcastle‐Ottawa Scale
OTU: operational taxonomic unit
OUD: opioid use disorder
PCoA: principal Coordinates Analysis
PEth: phosphatidylethanol
Pielou: Pielou's Evenness Index
PIENTER‐3: Peiling Immunisatie Effect Nederland ter Evaluatie van het Rijksvaccinatieprogramma (Survey of Immunization Effect in the Netherlands, third iteration)
PLCO: prostate, lung, colorectal, and ovarian cancer screening trial
PLWH: people living with HIV
PO: prescription opioids
qPCR: quantitative polymerase chain reaction
rRNA: ribosomal RNA
SAH: severe alcohol‐associated hepatitis
SCFA: short‐chain fatty acids
Shannon: Shannon index
Simpson: Simpson index
Sobs: observed species richness
spp.: species (plural, unspecified)
STI: sexually transmitted infections
SUD: substance use disorder
T1: timepoint 1 (at admission)
T2: timepoint 2 (after withdrawal treatment)
T2D: type 2 diabetes
TLFB: timeline followback
UniFrac: weighted/unweighted UniFrac distance
VHD: very heavy drinkers

## 1 Introduction

Substance use disorders (SUDs) represent a significant global health crisis, causing cycles of preventable harm, disability, and economic burden. Defined by the uncontrolled use of substances despite harmful consequences, SUDs involve symptoms, such as cravings, tolerance, withdrawal, and impaired functioning (Hasin et al. 2013). Globally, alcohol is a leading preventable risk factor, linked to over 200 medical conditions, including liver diseases, cancers, and injuries from accidents and violence (Lim et al. 2012; World Health Organization 2018). Cannabis, though rarely fatal, poses substantial harm due to its widespread use, while stimulant drug use continues to rise (United Nations: Office on Drugs and Crime, 2024). Among people who inject drugs, elevated risks of HIV and hepatitis C are prevalent, with liver diseases attributed to drug use accounting for over half of drug‐related deaths (United Nations: Office on Drugs and Crime, 2024). Opioids remain the primary contributors to drug‐related deaths and disability (United Nations: Office on Drugs and Crime, 2024). In the United States alone, 17.1% of people aged 12 or older had a SUD in 2023, including 10.2% with an alcohol use disorder (AUD), 9.6% with a drug use disorder, and 2.7% with both, highlighting the severe and overlapping consequences of substance misuse (SAMHSA, 2023).

Despite well‐established pharmacological treatments for some SUDs, such as AUD and opioid use disorder (OUD) (Rösner et al. 2010; Brewer et al. 2017; Mattick et al. 2009, 2014), their effectiveness remains limited. For instance, up to half of patients discontinue pharmacotherapy for OUD within 12 months (O'Connor et al. 2020). Furthermore, treatment options for other SUDs, such as stimulant or cannabis use disorders, remain insufficient (Ronsley et al. 2020; Castells et al. 2016; Pérez‐Mañá et al. 2013; Nielsen et al. 2019), and the effectiveness of psychosocial interventions varies significantly across conditions (Gates et al. 2016; Minozzi et al. 2024; Amato et al. 2011; Kaner et al. 2018; McQueen et al. 2011). These challenges underscore the need for deeper insights into the underlying mechanisms driving SUD pathophysiology and novel approaches to intervention.

In recent years, a neurobiological model has defined addiction as a chronic brain disorder with three stages: binge‐intoxication, withdrawal‐negative affect, and preoccupation‐anticipation (Koob and Volkow 2016). However, addiction is increasingly recognized as more than a brain‐centric condition, involving complex interactions between the central nervous system (CNS) and peripheral systems, including the digestive tract. The gut–brain axis, a bidirectional communication network connecting the CNS and gastrointestinal tract via neural, endocrine, immune, and humoral pathways, is shaped by the gut microbiome and plays a pivotal role in the development of various brain disorders, including mental health conditions (Cryan et al. 2019; Carabotti et al. 2015; Nikolova et al. 2021; Safadi et al. 2022) and addictive disorders (Hofford and Kiraly 2024; Chivero et al. 2022; Luo et al. 2023; Wang et al. 2022; Lucerne and Kiraly 2021; Barkus et al. 2024).

**FIGURE 1:**
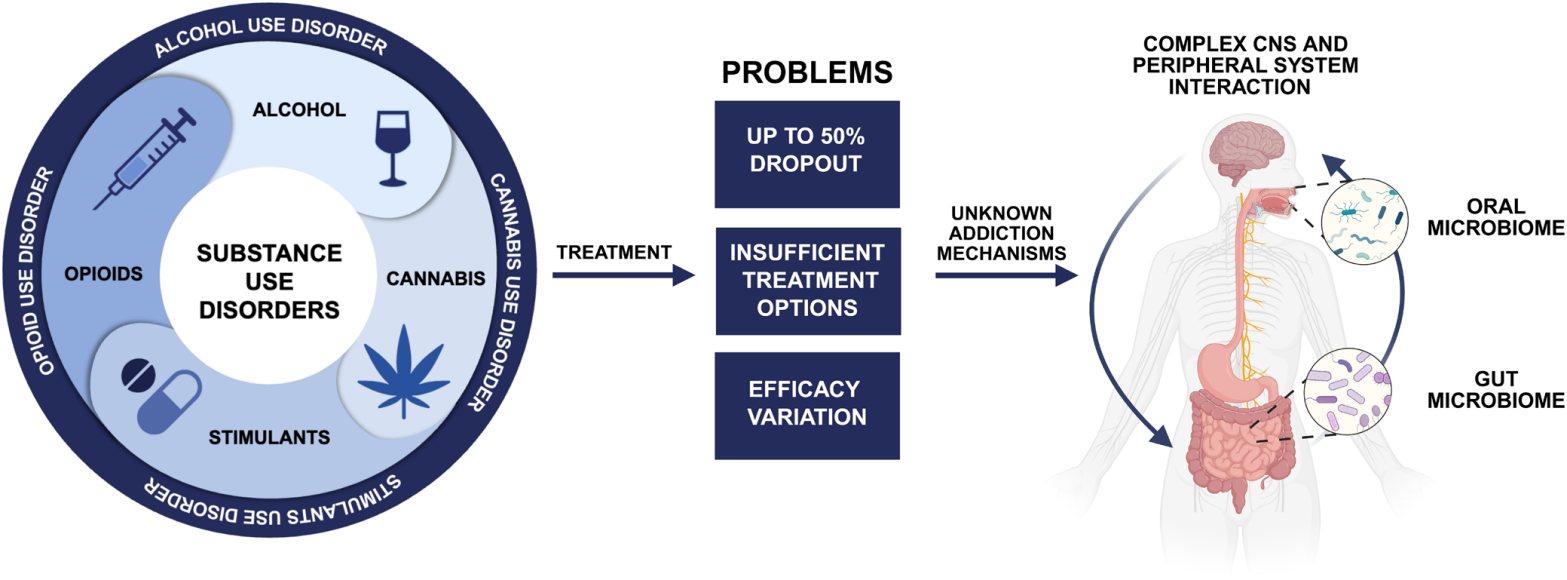
Schematic representation of substance use disorders, treatment challenges, and potential addiction mechanisms. CNS, central nervous system. Created in BioRender. Kazlauskaite, P. (2025) https://BioRender.com/zcyaxtb.

Although less studied than the gut microbiome, the oral microbiota plays an essential role in maintaining oral health, with dysbiosis implicated in periodontitis (Minty et al. 2019), systemic diseases, and mental health disorders (Bowland and Weyrich 2022; Peng et al. 2022; Lee et al. 2023; Lin et al. 2024; Manghi et al. 2024; Maitre et al. 2020). Recent findings suggest the oral microbiota may also influence the brain through mechanisms similar to those of the gut microbiota, including cranial nerve signaling and systemic circulation of microbes and their metabolites (Peng et al. 2022) (Figure 1).

Given these interactions, understanding the roles of the gut and oral microbiota in SUDs could offer novel insights into addiction mechanisms and therapeutic targets. This review focuses on human studies to explore the associations between microbiota and psychoactive substance use, synthesizing clinical evidence to clarify their role in SUD pathophysiology.

## 2 Methods

### 2.1 Literature Search Strategy

A comprehensive literature search was conducted using the PubMed database, with the final search performed on January 23, 2025. The following search query was used:

*(Gut Microbiota OR Gut Microbiome OR Oral Microbiota OR Oral Microbiome OR Dysbiosis) AND (“Alcohol Dependence” OR “Alcohol Abuse” OR* “Alcohol Use Disorder” *OR “Alcohol use” OR “Alcohol Addiction” OR “Alcohol Consumption” OR Cannabis OR Cannabinoids OR Marijuana OR Opioids OR Cocaine OR Methamphetamine OR Amphetamine OR Cathinones OR MDMA) NOT Review*.

The PubMed filter for human studies was applied. No time restriction was placed on publication dates. The inclusion criteria were:

1. Original clinical research articles on alcohol, opioid, stimulant, or cannabis use, including sequencing and analysis of the oral or gastrointestinal microbiome.
2. Studies presenting data on microbial composition, diversity, or function.
3. Studies enabling comparison between individuals who use substances (with or without SUDs) and control groups.
4. Peer‐reviewed articles written in English.

The exclusion criteria were:

1. Non‐original research (e.g., editorials, letters, commentaries, single‐case reports, conference abstracts, or meeting proceedings).
2. Reviews, systematic reviews, meta‐analyses, or other forms of secondary literature.
3. Non‐peer‐reviewed publications (e.g., preprints).
4. Studies that did not include any human participants (e.g., purely animal‐ or in vitro‐based microbiome experiments).
5. Studies lacking sequencing‐based or analytic data on the oral or gut microbiome in relation to psychoactive substance use.
6. Publications not available in English.

**FIGURE 2:**
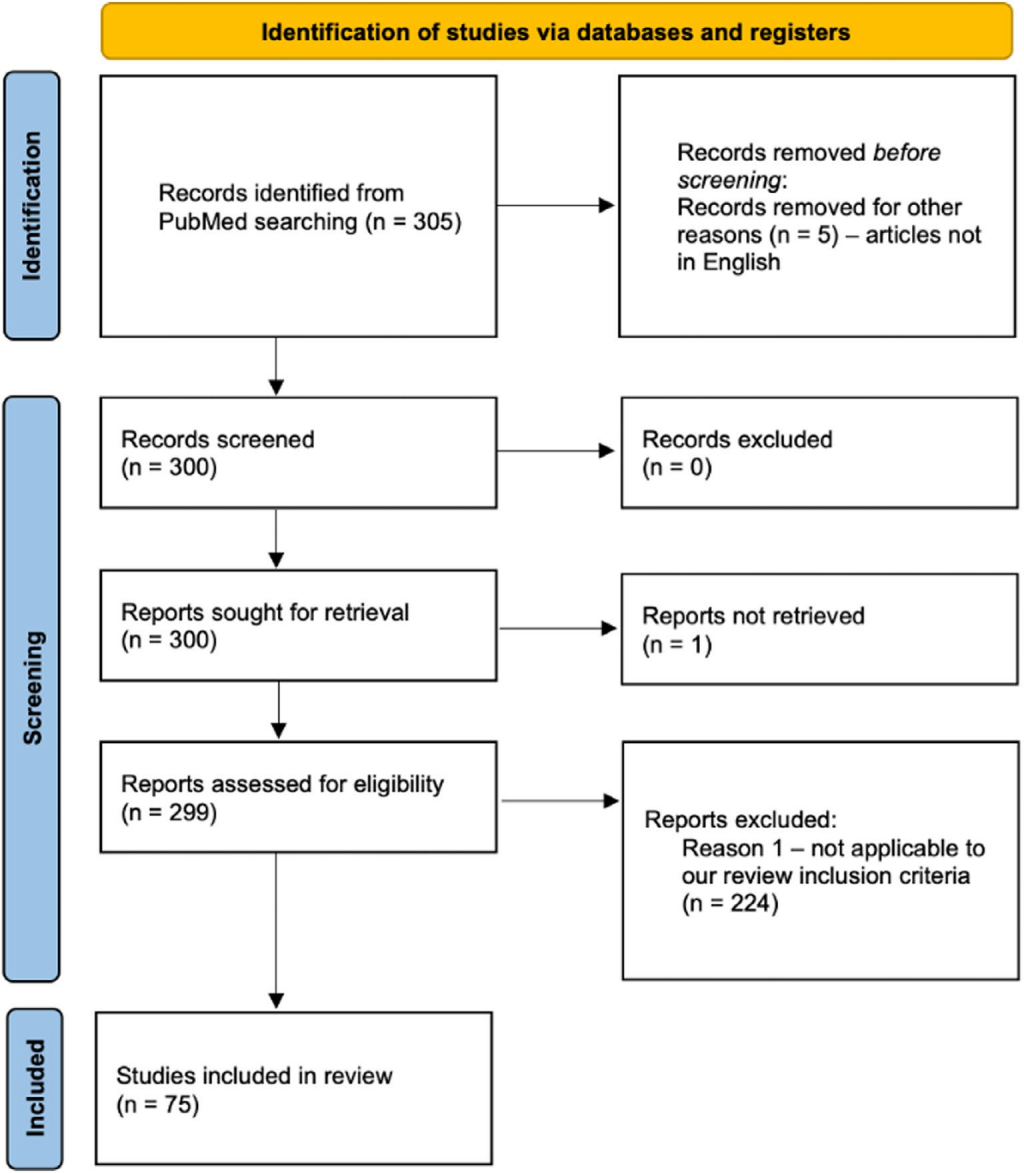
Literature search flow diagram.

Initially, articles were screened based on their titles and abstracts by two independent and experienced reviewers (A.B. and V.B.). Full‐text articles of relevant abstracts were retrieved and reviewed for inclusion. Additionally, a manual search of reference lists was conducted, and studies identified from these references that were relevant to our scope were also included to ensure comprehensiveness. All original clinical studies investigating the association between the oral or gut microbiome and psychoactive substance use were included in this scoping review (Figure 2). Risk‐of‐bias for each included study was assessed using the Newcastle‐Ottawa Scale (NOS); overall scores for all studies appear in Table S1. Institutional review board approval was not required.

## 3 Results

### 3.1 General Overview of Study Characteristics

**FIGURE 3:**
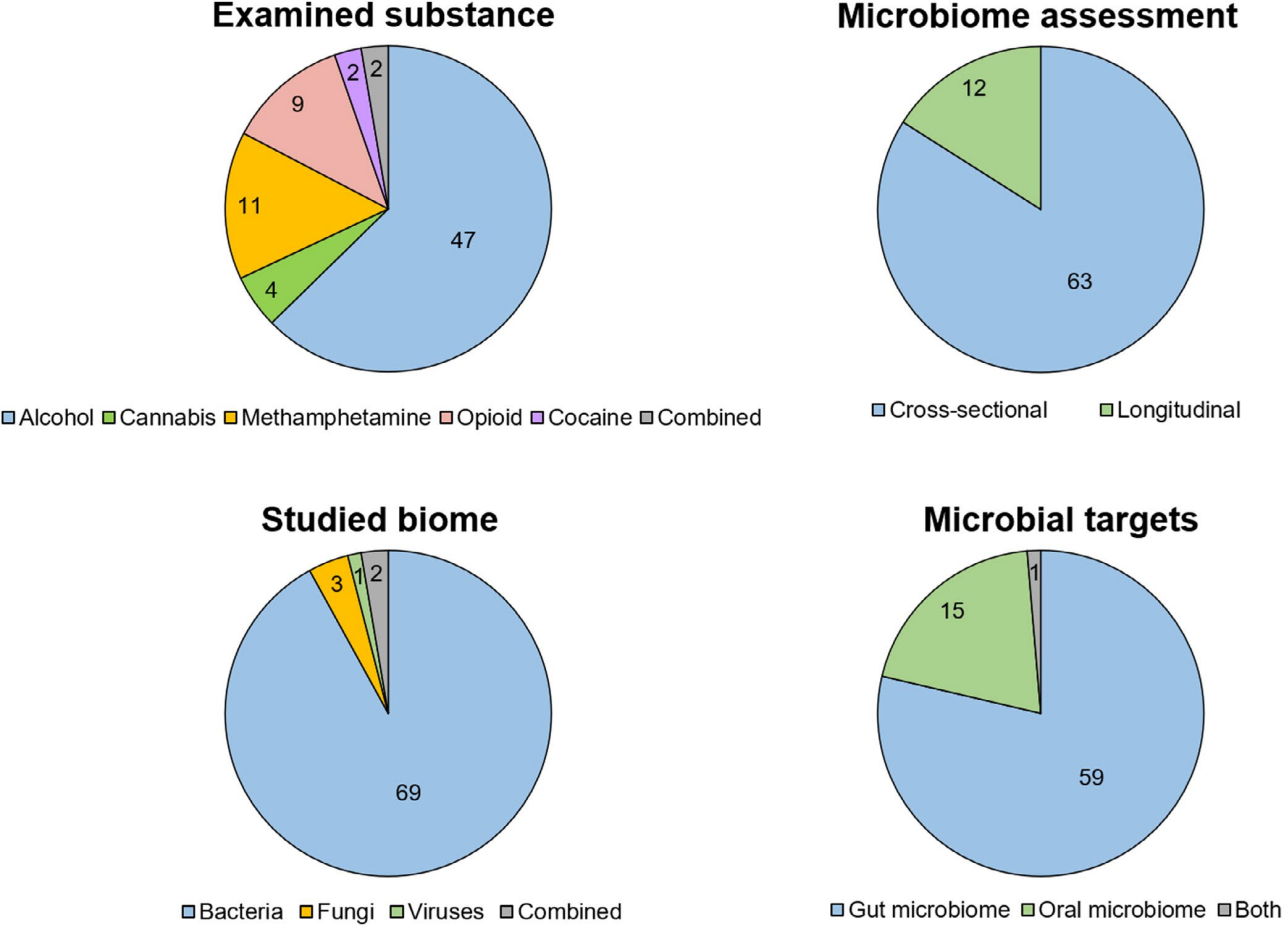
Number of reviewed studies by the examined substance, microbiome assessment, studied biome, and microbial targets.

**FIGURE 4:**
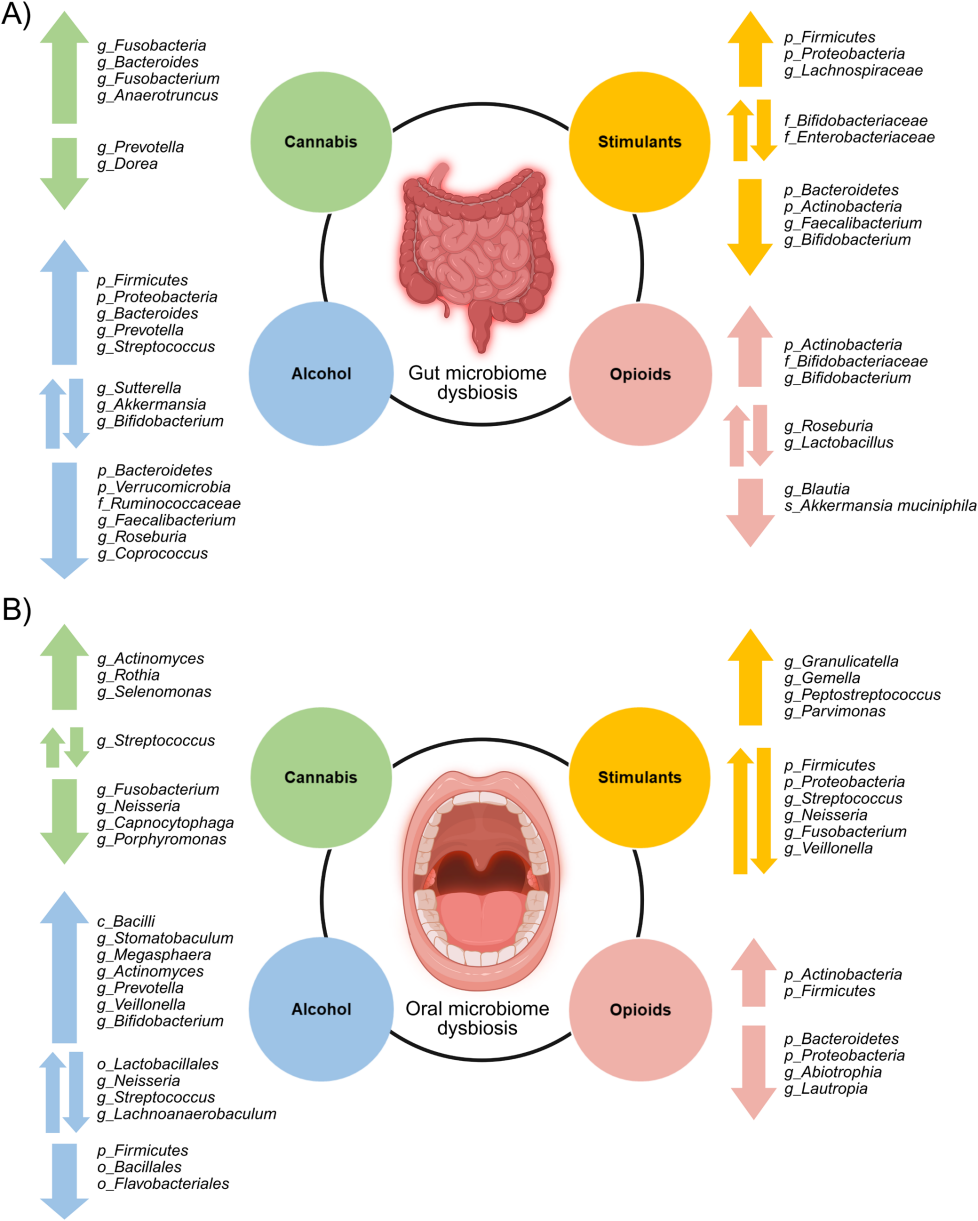
Summary of identified (A) gut and (B) oral bacteria across different substance use groups (alcohol, cannabis, stimulants, opioids) from the studies included in the review. f_family; g_genus; o_ order; P_ phylum; s_species. Created in BioRender. Kazlauskaite, P. (2025) https://BioRender.com/jto1hh2.

After a comprehensive literature search, 75 manuscripts were included that investigate the association between psychoactive substance use and changes in the gut or oral microbiota. Table 1 summarizes the data from these studies. Figure 3 presents the variety of studies by the examined substance, microbiome assessment, studied biome, and microbial targets. Figure 4 presents the summary of identified gut and oral bacteria across different substance use groups from the studies included in the review.

**TABLE 1:** A summary of the studies included in the review.

First author	Publication year	Substance	Biome	Study groups comparison	Sex	Microbiome assessment	Microbiome site and sampling method	Sequencing approach (hypervariable region)	Taxonomic identification method	Alpha diversity in relation to substance use	Alpha‐diversity measures	Beta‐diversity differences	Beta‐diversity distance metrics	Abundance differences?
Mutlu et al. (2012)	2012	Alcohol	Bacteria	HC (*n* = 18) vs. people with AUD (DSM‐IV) without (*n* = 29) and with liver disease (*n* = 19)	Male and Female	Cross‐sectional	Sigmoid colon mucosa biopsy	16S rRNA (V1‐V2)	OTU	No significant differences	Chao1	Yes	Weighted Unifrac	Yes
Kakiyama et al. (2014)	2014	Alcohol	Bacteria	103 subjects: HC (*n* = 19), active drinkers (*n* = 6), nonalcoholic cirrhosis (*n* = 30), abstinent AUD (unspecified criteria) patients (*n* = 38), active people with AUD with cirrhosis (*n* = 10). Stool microbiome comparison was made between **five active drinkers and five patients with nonalcoholic cirrhosis**	Male and Female	Cross‐sectional	Stool	16S rRNA (Region not specified)	OTU	NA	NA	NA	NA	Yes
Leclercq et al. (2014)	2014	Alcohol	Bacteria	Alcohol‐dependent patients (DSM‐IV) at admission (T1, *n* = 13) and after withdrawal treatment (T2, day 19)	Male and Female	Longitudinal	Stool	Pyrosequencing and qPCR of 16S rDNA	NA	NA	NA	NA	NA	Yes
Tsuruya et al. (2016)	2016	Alcohol	Bacteria	Patients with AUD (DSM‐IV) (*n* = 16) vs. HC (*n* = 48)	Male and Female	Cross‐sectional	Stool	16S rRNA (V1‐V2)	OTU	Increased	Chao1, ACE	Yes	Weighted Unifrac	Yes
Yang et al. (2017)	2017	Alcohol	Fungi	HC (*n* = 8), patients with AUD (unspecified criteria) without liver disease (*n* = 10), patients with alcoholic hepatitis (*n* = 6), patients with alcoholic liver cirrhosis (*n* = 4)	Male and Female	Cross‐sectional	Stool	ITS1 sequencing (Illumina MiSeq V2, primers BITS/B58S3)	OTU	Decreased	Simpson	NA	NA	Yes
Dubinkina et al. (2017)	2017	Alcohol	Bacteria	Patients with alcohol dependence (ICD‐10) (*n* = 72), patients with alcoholic cirrhosis (*n* = 27), and controls (*n* = 60)	Male and Female	Cross‐sectional	Stool	Shotgun metagenomic sequencing	NA	No significant differences	Shannon	Yes	Bray‐Curtis	Yes
Liao et al. (2018)	2018	Alcohol	Bacteria	About 47 participants classified as never (*n* = 30), former (*n* = 4; abstinent ≥ 6 months), or current (*n* = 12; daily drinking in past 6 months) drinkers	Male and Female	Cross‐sectional	Stool	16S rRNA (V3‐V4)	OTU	NA	NA	NA	NA	Yes
Kosnicki et al. (2019)	2019	Alcohol	Bacteria	Fecal samples from the American Gut Project: nondrinkers (*n* = 1495), regular drinkers (*n* = 1260) (3–5×/week), and daily drinkers (*n* = 692)	Male and Female	Cross‐sectional	Stool	16S rRNA (V4)	OTU	Decreased	Faith's Phylogenetic Diversity, Chao1	NA	Unweighted Unifrac	Yes
Lang et al. (2020)	2020	Alcohol	Fungi	Patients with alcoholic hepatitis (*n* = 59), patients with AUD (DSM‐IV) (*n* = 15), controls (*n* = 11)	Male and Female	Cross‐sectional	Stool	ITS1 sequencing (Illumina MiSeq V2, primers BITS/B58S3)	NA	Decreased	Shannon, Chao1, Simpson	Yes	Euclidean distance metric	Yes
Zhao et al. (2020)	2020	Alcohol	Bacteria	Patients with alcohol dependence (ICD‐10) (*n* = 3), alcohol‐free controls (*n* = 3)	Male	Cross‐sectional	Stool	16S rRNA (V3‐V5)	OTU	No significant differences	Shannon, Chao1, Simpson	NA	NA	Yes
Bjørkhaug et al. (2019)	2019	Alcohol	Bacteria	Alcohol overconsumption (*n* = 24, > 20/40 g/day for women/men, > 10 years), controls (*n* = 18, < 5/10 g/day for women/men)	Male and Female	Cross‐sectional	Stool	16S rRNA (V3‐V4)	OTU	No significant differences	Shannon	No	Weighted and Unweighted Unifrac	Yes
Duan et al. (2019)	2019	Alcohol	Bacteria	Social drinkers (*n* = 26; < 20 g/day), patients with AUD (DSM‐IV) (*n* = 44; > 60 g/day), and patients with alcoholic hepatitis (*n* = 88)	Male and Female	Cross‐sectional	Stool	16S rRNA (V4)	OTU	Decreased	Shannon, Chao1, Simpson	Yes	Jaccard dissimilarity matrices	NA
Seo et al. (2020)	2020	Alcohol	Bacteria	Three groups based on AUDIT score: Zone I (*n* = 165; 0–7), Zone II (*n* = 130; 8–15), Zone III (*n* = 115; 16–40)	Male and Female	Cross‐sectional	Stool	16S rRNA (V3–V4)	OTU	Decreased (zone III)	Shannon	NA	NA	Yes
Addolorato et al. (2020)	2020	Alcohol	Bacteria	Patients with AUD (DSM‐5) (*n* = 36) vs. healthy low‐risk controls (*n* = 36)	Male and Female	Cross‐sectional	Stool	16S rRNA (V3–V4)	OTU	Decreased	Shannon	Yes	Weighted Unifrac	Yes
Rodríguez‐Rabassa et al. (2020)	2020	Alcohol	Bacteria	Participants with AUDIT score ≥ 8 (*n* = 30) vs. controls (*n* = 20)	Male and Female	Cross‐sectional	Saliva	16S rRNA (V3–V4)	OTU	No significant differences	Shannon	No	Bray‐Curtis	Yes
Smirnova et al. (2020)	2020	Alcohol	Bacteria	HC (*n* = 20), heavy drinking controls (> 5 units/day) (*n* = 20), mild alcoholic hepatitis (*n* = 10), severe alcoholic hepatitis (*n* = 24)	Male and Female	Cross‐sectional	Stool	16S rRNA (V1–V2)	OTU	No significant differences	Shannon	Mixed	Bray‐Curtis	Yes
Adams et al. (2020)	2020	Alcohol	Bacteria	Patients undergoing liver biopsy with/without NAFLD: alcohol consumers (*n* = 40) vs. nonconsumers (*n* = 49; < 3 standard drinks/day)	Male and Female	Cross‐sectional	Stool	16S rRNA (V6–V8)	OTU	NA	NA	NA	NA	Yes
Maccioni et al. (2020)	2020	Alcohol	Bacteria	HC (*n* = 24) vs. patients with AUD (DSM‐IV) (*n* = 106) admitted for 3‐week withdrawal treatment. All AUD patients consumed > 60 g/day for > 1 year and were actively drinking until admission	Male and Female	Longitudinal	Duodenal mucosa; Stool	16S rRNA (Region not specified)	OTU	**Duodenum**: No significant differences **Gut**: Decreased **After 3 weeks**: No significant differences	Observed OTUs, Chao1, Shannon, Simpson	Yes	Weighted and Unweighted Unifrac	Yes
Ames et al. (2020)	2020	Alcohol	Bacteria	Less heavy drinkers (LHD; *n* = 8; < 10 drinks/day) vs. very heavy drinkers (VHD; *n* = 14; ≥ 10 drinks/day). All met AUD criteria (Alcohol Dependence Scale > 9)	Male and Female	Longitudinal	Saliva, stool	16S rRNA (V2‐4‐8 and V3‐67‐9)	OTU	No significant differences	Shannon	NA	NA	Yes
Jiang et al. (2020)	2020	Alcohol	Viruses, Bacteria	Patients with alcoholic hepatitis (*n* = 89), patients with AUD (DSM‐IV) (*n* = 36), and controls (*n* = 17)	Male and Female	Cross‐sectional	Stool	Bacterial: 16S rRNA (V4) Virome: NetoVIR, Illumina sequencing, PathSeq analysis	Whole‐genome alignment (PathSeq)	Increased	Shannon, inversed Simpson, Chao1	NA	NA	Yes
Maffei et al. (2021)	2021	Alcohol	Bacteria	359 PLWH participants assessed for alcohol use via self‐report (AUDIT, TLFB) and PEth	Male and Female	Cross‐sectional	Stool	16S rRNA (V4)	ASV	No significant differences	Total number of denoised, unique sequence variants observed per sample	Yes	Generalized UniFrac distance	Yes
Lin et al. (2020)	2020	Alcohol	Bacteria	Nondrinkers (*n* = 14), smoking‐only (*n* = 31), drinking‐only (*n* = 28), and smoking + drinking (*n* = 43) participants	Male	Cross‐sectional	Stool	16S rRNA (V3–V4)	OTU	No significant differences	Shannon, Sobs, Heip	Yes	Bray‐Curtis (PCoA)	Yes
González‐Zancada et al. (2020)	2020	Alcohol	Bacteria	Abstainers or < 1.5 g alcohol/day (*n* = 44) vs. beer consumers (*n* = 34; ≥ 70% total alcohol from beer, 10–30 g/day, minimal wine/spirits)	Male and Female	Cross‐sectional	Stool	16S rRNA (V3–V4)	OTU	No significant differences	Shannon, Chao1	No	Bray‐Curtis (PCoA)	Yes
Gurwara et al. (2020)	2020	Alcohol	Bacteria	Never (ND, *n* = 9), former (FD, *n* = 10), light (LD, < 2 drinks/day, *n* = 9), heavy (HD, > 48 g/day, *n* = 6) drinkers	Male and Female (*N* = 1)	Cross‐sectional	Colonic mucosa biopsy	16S rRNA (V4)	OTU	Decreased (HD)	Shannon, Simpson	No	Weighted Unifrac	Yes
Vujkovic‐Cvijin et al. (2020)	2020	Alcohol	Bacteria	American Gut Project samples (*n* = 350; 175 drinkers, 175 nondrinkers) matched on confounders, validated with external cohorts (*n* = 70)	Male and Female	Cross‐sectional	Stool	16S rRNA (V4)	ASV	Increased	Shannon	NA	NA	Yes
Kim et al. (2020)	2020	Alcohol	Bacteria	38 CRC patients (alcohol drinker/nondrinker: *n* = 15/17) and 21 controls (drinker/nondrinker: *n* = 10/6). Classified as non/light (0–1 drink/day) or heavy (≥ 2 drinks/day) drinkers	Male and Female	Cross‐sectional	Colonic mucosa biopsy	16S rRNA (V4)	OTU	No data on relation to alcohol use	No data on relation to alcohol use	No data on relation to alcohol use	No data on relation to alcohol use	Yes
Wang et al. (2021)	2021	Alcohol	Bacteria	Late‐pregnancy women with (*n* = 10) vs. without (*n* = 19) alcohol use; infant fecal samples collected within 48 h of birth	Female (pregnant women) and their newborns (male and female)	Cross‐sectional	Stool	16S rRNA (V3‐V4)	OTU	Mothers: Increased Newborns: Increased	Shannon	Yes	Unweighted Unifrac, abund‐Jaccard	Yes
Kwan et al. (2022)	2022	Alcohol	Bacteria	Subjects from Cameron County Hispanic Cohort (*n* = 217): never (*n* = 145), moderate (*n* = 62), heavy (*n* = 10; > 10/20 g/day for women/men) drinkers	Male and Female	Cross‐sectional	Stool	16S rRNA (V4); Shotgun metagenomic sequencing	OTU	No data on relation to alcohol use	No data on relation to alcohol use	Yes	Weighted Unifrac	No data on relation to alcohol use
Hsu et al. (2022)	2022	Alcohol	Viruses	Patients with AUD (DSM‐IV) (*n* = 62; > 60 g/day for > 1 year) admitted for 3‐week withdrawal treatment vs. HC (*n* = 16)	Male and Female	Longitudinal	Stool	Bacterial: 16S rRNA (V4) Virome: NetoVIR, Illumina sequencing, PathSeq analysis	Whole‐genome alignment (PathSeq)	NA	NA	Yes	Bray‐Curtis (PCoA)	Yes
Hoang et al. (2023)	2023	Alcohol	Bacteria	CRC patients (*n* = 331): ever (*n* = 126) vs. never (*n* = 205) consumed alcohol	Male and Female	Cross‐sectional	Stool	16S rRNA (V3–V4)	OTU	No data on relation to alcohol use	No data on relation to alcohol use	No data on relation to alcohol use	No data on relation to alcohol use	Yes
Carbia et al. (2023)	2023	Alcohol	Bacteria	Young adults (*n* = 75; aged 18–25). Alcohol use measured with AUDIT and TLFB. Categorized into tertiles: low drinkers, binge drinkers (BDs), high BDs	Male and Female	Cross‐sectional	Stool	Shotgun metagenomic sequencing	NA	No significant differences	Shannon Entropy, Chao1, Simpson	Yes	Aitchison distance, Euclidean distance	Yes
Szóstak et al. (2023)	2023	Alcohol	Fungi	Participants from the Polish Microbiome Map (*n* = 923): never (*n* = 206), ever (*n* = 716)—rarely (*n* = 130), monthly (*n* = 213), weekly (*n* = 338), daily (*n* = 31) drinkers	Male and Female	Cross‐sectional	Stool	Whole‐metagenome sequencing	OTU	Mixed results varied by group comparison: Alcohol ever vs. Never: Decreased Alcohol daily and weekly vs. Monthly: Increased	Fungal richness (species count), evenness (Pilou evenness), and effective diversity (Shannon's index, diversity function)	Yes	Jaccard distance	Yes
Zhao et al. (2023)	2023	Alcohol	Bacteria	Patients with alcohol dependence (DSM‐IV) (*n* = 32) vs. controls (*n* = 20)	Male	Cross‐sectional	Stool	16S rRNA (V4)	OTU	Decreased	Chao1	Yes	Weighted Unifrac	Yes
Kyaw et al. (2023)	2023	Alcohol	Bacteria	CRC survivors (*n* = 28) with alcohol use correlation data	Male and Female	Cross‐sectional	Stool	16S rRNA (V4)	ASV	Increased	Shannon	Yes	Weighted Unifrac	Yes
Philips et al. (2023)	2023	Alcohol	Bacteria	Severe alcohol‐associated hepatitis (SAH; *n* = 37), divided into FMT (*n* = 16) and corticosteroid treatment (*n* = 14) groups followed for at least 12 months Significant daily drinking: > 2 drinks/day (men) or > 1 (women); binge: ≥ 4 (women)/≥ 5 (men) in 2 h at least once in last 30 days	Male	Longitudinal	Stool	16S rRNA (V3‐V4)	ASV	No significant differences	Shannon	Yes	Bray‐Curtis, Weighted UniFrac	Yes
Wang, Yan, et al. (2023)	2023	Alcohol	Bacteria + Fungi	AUD patients (DSM‐5) (*n* = 34) vs. HC (*n* = 19). 24 fecal samples used for metabolome analysis (AUD = 14, control = 10)	Male	Cross‐sectional	Stool	16S rRNA (V3‐V4); ITS1 sequencing	ASV	No significant differences	Shannon, Chao1, Simpson	Yes	Unweighted Unifrac (bacteria), Bray‐Curtis (Fungi)	Yes
Qiao et al. (2024)	2024	Alcohol	Bacteria	93 PLWH (≥ 18 years): low/moderate drinkers (*n* = 21; < 70–210 g/week for men, < 70–140 for women) vs. nondrinkers (*n* = 72)	Male and Female	Cross‐sectional	Stool	16S rRNA (V4)	OTU	No significant differences	Shannon, Chao1	No	Weighted and Unweighted Unifrac	Yes
Hoisington et al. (2024)	2024	Alcohol	Bacteria	Burn patients (*n* = 19) categorized by PEth levels: low (< 20 ng/mL, *n* = 12; 0% AUD) vs. high (≥ 20 ng/mL, *n* = 7; 100% AUD by AUDIT‐C)	Male and Female	Cross‐sectional	Stool	16S rRNA (V3‐V4)	ASV	No significant differences	Observed Features, Shannon, Pielou	No	Weighted and Unweighted Unifrac	Yes
Piacentino et al. (2024)	2024	Alcohol	Bacteria	Three groups: abstinent individuals with AUD (DSM‐5) (*n* = 10) after ≥ 4 weeks inpatient treatment and abstinent for ≥ 2 weeks, currently drinking individuals with AUD (*n* = 9; NIAAA criteria for heavy drinking), HC (*n* = 12; no AUD, ≤ 1/2 drink/day for women/men)	Male and Female	Cross‐sectional	Stool	16S rRNA (V3‐V4)	ASV	Increased in CD (Chao1, Observed ASVs) No differences in Shannon and Simpson indices	Observed ASVs, Chao1, Shannon, Simpson	No	Bray‐Curtis	Yes
Wang, Pan, et al. (2024)	2024	Alcohol	Bacteria	GWAS data for gut microbiota and alcohol abuse from MiBioGen and Finngen databases (18 340 participants across 16 cohorts, 18 countries)	Male and Female	Cross‐sectional	Stool	Multivariable Mendelian randomization analysis of sequencing profiles of the 24S ribosomal RNA gene and genetic typing data	NA	NA	NA	NA	NA	Yes
Li et al. (2024)	2024	Alcohol	Bacteria	Healthy participants (*n* = 39) vs. patients with alcohol dependence (unspecified criteria) (*n* = 33)	Male and Female	Cross‐sectional	Stool	16S rRNA (V3‐V4)	NA	Decreased	Shannon	Yes	Unweighted Unifrac	Yes
Börnigen et al. (2017))	2017	Alcohol	Bacteria	Patients with squamous cell carcinoma of the oral cavity or oropharynx vs. HC. Alcohol use: never (*n* = 8), never/former regular (*n* = 187), current (*n* = 168; ≥ 1 drink/month, median 8 drinks/week, mean 22 drinks/week) users	Male and Female	Cross‐sectional	Saliva	16S rRNA (V4)	OTU	No data on relation to alcohol use	No data on relation to alcohol use	No data on relation to alcohol use	No data on relation to alcohol use	Yes
Fan et al. (2018)	2018	Alcohol	Bacteria	Participants from the ACS CPS II cohort and the NCI PLCO cohort Nondrinkers (*n* = 270), moderate drinkers (*n* = 614, ≤ 1/2 drinks/day for women/men), heavy drinkers (*n* = 160, > 1/2 drinks/day for women/men)	Male and Female	Cross‐sectional	Mouthwash	16S rRNA (V3‐V4)	OTU	Increased	Observed species, inversed Simpson	Yes	Unweighted Unifrac	Yes
Ortiz et al. (2022)	2022	Alcohol and Marihuana	Bacteria	134 Hispanic adults (aged 21–49) from STI clinics: alcohol use in past 12 months (yes = 81, no = 14); marijuana use (yes = 66, no = 68)	Male and Female	Cross‐sectional	Saliva	16S rRNA (V4)	OTU	Alcohol: Increased Marihuana: No significant differences	Shannon, Chao1	Alcohol: No Marihuana: No	Bray‐Curtis	Yes
Ward et al. (2023)	2023	Alcohol	Bacteria	Heavy drinkers (*n* = 11) vs. light drinkers (*n* = 12) based on NIH binge drinking criteria	Male and Female	Cross‐sectional	Saliva	16S rRNA (V4) and metagenomic sequencing	ASV	No significant differences	Shannon, Inversed Simpson's, Observed Species	No	Bray‐Curtis	Yes
Yadav et al. (2023)	2023	Alcohol	Bacteria	Healthy volunteers including alcohol‐dependent patients (unspecified), nonusers (50 per category). Alcoholic participants consumed ≥ 180 mL at night before sampling	Not specified	Cross‐sectional	Saliva	16S rRNA (V3‐V4)	ASV	NA	NA	NA	NA	Yes
Maley et al. (2024)	2024	Alcohol	Bacteria	Postmenopausal women (*n* = 1179) Nondrinkers (*n* = 393), < 1 drink/week (*n* = 273); drinking 1–< 7 times/week (*n* = 352), drinking ≥ 7 times/week (*n* = 161). High‐intensity drinkers ≥ 2 drinks/occasion	Female	Cross‐sectional	Saliva	16S rRNA (V3‐V4)	OTU	Increased	Shannon, Chao1	yes	Euclidean distance metric	Yes
Odendaal et al. (2024)	2024	Alcohol	Bacteria	3104 saliva samples of 3160 Dutch individuals 0–87 years of age, participating in a cross‐sectional population‐wide study (PIENTER‐3): Alcohol drinkers (*n* = 1528), nondrinkers (*n* = 529), unknown (*n* = 1103)	Male and Female	Cross‐sectional	Saliva	16S rRNA (V4)	ASV	Increased	Shannon	No data on relation to alcohol use	No data on relation to alcohol use	Yes
Morgan et al. (2024)	2024	Cannabis	Bacteria	Young sexual/gender minorities (*n* = 42): never used cannabis (*n* = 16), intermittent use (*n* = 10; ≤ 5 times/30 days), frequent use (*n* = 16; ≥ 6 times/30 days). Cannabis use also assessed by CUDIT	Male	Cross‐sectional	Rectal swab	16S rRNA (V4)	ASV	Decreased (problematic use)	Shannon, Pielou	No	Not specified	Yes
Panee et al. (2018)	2018	Cannabis	Bacteria	Chronic marijuana users (*n* = 19; ≥ 3 times/week for ≥ 3 years) vs. nonusers (*n* = 20; < 10 lifetime uses, no recent use)	Male and Female	Cross‐sectional	Stool	16S rRNA (region not specified)	ASV	NA	NA	NA	NA	Yes
Newman et al. (2019)	2019	Cannabis	Bacteria	Frequent marijuana users (*n* = 20; 17 of them—daily/almost daily) vs. age/gender‐matched controls (*n* = 19; no marijuana/tobacco)	Male and Female	Cross‐sectional	Tongue and oral pharynx swabs	16S rRNA (V1‐V3)	OTU	No significant differences	Shannon, Pielou, Margalef	No	Bray‐Curtis	Yes
Luo et al. (2021)	2021	Cannabis	Bacteria	Chronic cannabis smokers with CUD (DSM‐5) (*n* = 16) vs. nonsmoking controls (*n* = 27)	Male and Female	Cross‐sectional	Saliva	16S rRNA (V3‐V4)	OTU	Decreased	Shannon	Yes	Bray‐Curtis	Yes
Martinez et al. (2022)	2022	Cocaine	Bacteria	PLWH who used cocaine (*n* = 25; ≥ 1 year consistent use) vs. PLWH who did not (*n* = 25)	Male and Female	Cross‐sectional	Stool	16S rRNA (V4)	ASV	No significant differences	Observed ASV richness, Shannon, Simpson	No	UniFrac (unspecified)	Yes
Fu et al. (2022)	2022	Cocaine	Bacteria	Individuals with cocaine use disorder (DSM‐5) (*n* = 8) vs. healthy nonusers (*n* = 10)	Male and Female	Cross‐sectional	Saliva	16S rRNA (V4)	OTU	Decreased	Shannon, Simpson	NA	NA	Yes
Fulcher et al. (2018)	2018	Methamphetamine and Cannabis	Bacteria	PLWH (*n* = 37, MSM) at two time points (T1 and T2), separated by 6 months. MA use T1: *n* = 24, T2: *n* = 21; Cannabis use T1: *n* = 18, T2: *n* = 11	Male	Longitudinal	Rectal swab	16S rRNA (V1‐V2)	ASV	No significant differences	Not specified	Yes	Bray‐Curtis	Yes
Cook et al. (2019)	2019	Methamphetamine	Bacteria	Methamphetamine (MA) users (*n* = 156; any use in the last 6 months) vs. nonusers (*n* = 225), all MSM	Male	Cross‐sectional	Rectal swab	16S rRNA (V4)	ASV	No significant differences	Shannon, Chao1, Simpson	Yes	Bray‐Curtis, Jaccard, Jensen‐Shannon	Yes
Yang, Yu, Liu, et al. (2021)	2021	Methamphetamine	Bacteria	Subjects with MUD (DSM‐5) (*n* = 16) vs. HC (*n* = 14)	Male	Cross‐sectional	Stool	16S rRNA (V4)	OTU	Decreased (Shannon)	Shannon, Chao1, Observed species	Yes	Bray‐Curtis, Weighted and Unweighted UniFrac	Yes
Deng et al. (2021)	2021	Methamphetamine	Bacteria	MUD patients (DSM‐5) (*n* = 26) vs. healthy individuals (*n* = 17)	Male	Cross‐sectional	Stool	16S rRNA (V3‐V4)	OTU	No significant differences	Shannon, Chao1, Observed species, Simpson	No	Bray‐Curtis, Jaccard, Weighted and Unweighted UniFrac	Yes
Wang, Wang, et al. (2023)	2023	Methamphetamine	Bacteria	METH group (ICD‐10) (*n* = 139) vs. HC (*n* = 84)	Male and Female	Longitudinal	Stool	16S rRNA (V3‐V4)	OTU	Decreased	Observed species, Chao1, Shannon, Simpson, ACE, and good coverage	Yes	Bray‐Curtis	Yes
He et al. (2023)	2023	Methamphetamine	Bacteria	Casual MA users (*n* = 21; MCU; 0 or 1 DSM‐5 criteria) vs. people with MUD (*n* = 45; ≥ 2 of 11 DSM‐5 criteria)	Male and Female	Cross‐sectional	Stool	16S rRNA (V4)	ASV	No significant differences	Faith, Shannon, observed features, Pielou	No	Bray‐Curtis, Jaccard, Weighted and Unweighted UniFrac	Yes
Liu et al. (2023)	2023	Methamphetamine	Bacteria	MUD (DSM‐5) (*n* = 78) vs. HC (*n* = 50)	Male and Female	Cross‐sectional	Stool	16S rRNA (V3‐V4)	ASV	Decreased	Shannon, Chao1	Yes	Bray‐Curtis	Yes
Liu et al. (2024)	2024	Methamphetamine	Bacteria	MUD (unspecified criteria) patients (*n* = 62) vs. HC (*n* = 50) 25 MUD patients followed for 2 months post‐abstinence	Male and Female	Longitudinal	Stool	16S rRNA (V3‐V4)	ASV	Decreased	ACE, Chao1, Shannon, Simpson	Yes	Bray‐Curtis	Yes
Deng et al. (2024)	22 024	Methamphetamine	Bacteria	MA users (DSM‐5) (*n* = 70) vs. HC (*n* = 38; all good sleep). Subgroups: MA‐GS (good sleep, *n* = 49), MA‐BS (bad sleep, *n* = 21)	Male and Female	Cross‐sectional	Stool	16S rRNA (V3‐V4)	ASV	No significant differences	ACE, Chao1, Shannon, Simpson	Yes	Bray‐Curtis	Yes
Yang, Yu, Yang, et al. (2021)	2021	Methamphetamine	Bacteria	MUD patients (unspecified criteria) (*n* = 20) vs. HC (*n* = 14), plus 12 MA users after 2‐week abstinence	Male	Longitudinal	Saliva	16S rRNA (V4)	OTU	Decreased (Chao1, ACE) 2 weeks: No significant differences	ACE, Chao1, Shannon, Simpson	Yes	Weighted and Unweighted Unifrac	Yes
Deng et al. (2022)	2022	Methamphetamine	Bacteria	People with MUD (unspecified criteria) (*n* = 30) vs. HC (*n* = 15)	Female	Cross‐sectional	Dental plaque	16S rRNA (V3‐V4)	ASV	No significant differences	Chao1, observed species, Shannon, Simpson, Faith's PD, Pielou's evenness, Good's coverage	Yes	Weighted and Unweighted Unifrac	Yes
Wang, Feng, et al. (2024)	2024	Methamphetamine	Bacteria	MUD (unspecified criteria) patients (*n* = 278) vs. HC (*n* = 105)	Male and Female	Cross‐sectional	Saliva	16S rRNA (V3‐V4)	OTU	Decreased	ACE, Chao1, Shannon, Simpson	Yes	Jaccard distance	Yes
Acharya et al. (2017)	2017	Opioid	Bacteria	Cirrhotic outpatients on chronic opioids (*n* = 72) vs. age/MELD‐matched cirrhotic controls (*n* = 72) not on opioids	Male and Female	Cross‐sectional	Stool	16S rRNA (V1‐V2)	OTU	NA	NA	Yes	UniFrac (unspecified)	Yes
Barengolts et al. (2018)	2018	Opioid	Bacteria	African American men (*n* = 99), 45 with OUD (DSM‐IV)	Male	Cross‐sectional	Stool	16S rRNA (V3‐V4)	OTU	No data on relation to opioid use	Shannon	No data on relation to opioid use	Bray‐Curtis	Yes
Pettigrew et al. (2019)	2019	Opioid	Bacteria	ICU patients (*n* = 109), 80 receiving opioids	Male and Female	Cross‐sectional	Perirectal swab	16S rRNA (V4)	OTU	No data on relation to opioid use	Shannon, Simpson	NA	NA	Yes
Li et al. (2020)	2020	Opioid	Bacteria	CDet patients (*n* = 28), MMT patients (MP, *n* = 16), current DU participants with heroin or MA use disorders (*n* = 27) and HCs (*n* = 28)	Male and Female	Cross‐sectional	Stool	16S rRNA (V3‐V4)	OTU	No significant differences	Observed species, Chao1, Shannon, Simpson, ACE, and excellent coverage	Yes	Jaccard distance	Yes
Gicquelais et al. (2020)	2020	Opioid	Bacteria	Patients receiving outpatient addiction (unspecified criteria) treatment: (*n* = 46): **Ag** only (heroin/PO, *n* = 5), **AgAt** (buprenorphine–naloxone + PO + naltrexone, *n* = 4), **At** (naltrexone only, *n* = 6), **N** (neither Ag nor At, *n* = 31); 3 samples weekly	Male and Female	Longitudinal	Stool	16S rRNA (V4)	Oligotyping	Alpha diversity: decreased (Ag vs. N) No significant differences (AgAt and At vs. N)	Shannon, Chao1	No	Aitchison distance	Yes
Cruz‐Lebrón et al. (2021)	2021	Opioid	Bacteria	Non‐opioid users (*n* = 28) vs. methadone‐treated individuals (*n* = 34)	Male and Female	Cross‐sectional	Stool	16S rRNA (V3‐V4)	OTU	Decreased	Shannon, Chao1	Yes	Not specified	Yes
Nguyen et al. (2023)	2023	Opioid	Bacteria	About 9167 fecal samples from 1201 allo‐HCT patients with recorded drug exposures	Male and Female	Longitudinal	Stool	16S rRNA (V4‐V5) and shotgun metagenomic sequencing	ASV	NA	NA	NA	NA	Yes
Xie et al. (2024)	2024	Opioid	Bacteria	Individuals with OUD (unspecified criteria) (*n* = 58; heroin = 7, morphine = 50, fentanyl = 1) vs. HC (*n* = 50)	Not specified	Longitudinal	Stool	16S rRNA (V3–V4)	OTU	No data on relation to opioid use	Observed species, Chao1, Shannon, Simpson, ACE, and excellent coverage	Yes	Bray‐Curtis	Yes
Wu et al. (2021)	2021	Opioid	Bacteria	Participants (*n* = 558): never users (*n* = 373), cigarette‐only (*n* = 120), opium‐only (*n* = 16), both (*n* = 49)	Male and Female	Cross‐sectional	Saliva	16S rRNA (V4)	ASV	Decreased	Observed ASVs, Shannon, Faith's PD	Yes	Bray‐Curtis, Weighted and Unweighted UniFrac	Yes

Abbreviations: ACE, abundance‐based coverage estimator; ACS, American Cancer Society; AD, alcohol dependence; Ag, opioid agonist; AgAt, opioid agonist and antagonist; Allo‐HCT, allogeneic hematopoietic cell transplantation; ASV, amplicon sequence variant; At, opioid antagonist; AUD, alcohol use disorder; AUDIT, alcohol use disorders identification Test; AUDIT‐C, short version of AUDIT; BDs, binge drinkers; Bray–Curtis, Bray–Curtis dissimilarity; CD, current drinkers; CDet, compulsory detention patients; Chao1, Chao1 index; CPS II, Cancer Prevention Study II; CRC, colorectal cancer; CUD, cannabis use disorder; CUDIT, cannabis use disorder identification test; DSM‐5, Diagnostic and Statistical Manual of Mental Disorders, Fifth Edition; DSM‐IV, Diagnostic and Statistical Manual of Mental Disorders, Fourth Edition; DU, drug users; Faith's PD, Faith's phylogenetic diversity; FD, former drinkers; FMT, fecal microbiota transplantation; GWAS, genome‐wide association study; HC, healthy controls; HD, heavy drinkers; HDC, heavy drinking controls; Heip, Heip's evenness index; ICD‐10, International Classification of Diseases, Tenth Revision; ICU, intensive care unit; ITS1, internal transcribed spacer 1; Jaccard, Jaccard distance; Jensen–Shannon, Jensen–Shannon distance; LD, light drinkers; LHD, less heavy drinkers; MA, methamphetamine; Margalef, Margalef richness index; MELD, model for end‐stage liver disease; MMT, methadone maintenance therapy; MP, MMT patients; MSM, men who have sex with men; MUD, methamphetamine use disorder; N, neither opioid agonist nor antagonist; NA, not applicable/not available; NAFLD, nonalcoholic fatty liver disease; NCI, National Cancer Institute; ND, never drinkers; NetoVIR, NetoVIR viral metagenomics pipeline; NIAAA, National Institute on Alcohol Abuse and Alcoholism; NIH, National Institutes of Health; OTU, operational taxonomic unit; OUD, opioid use disorder; PCoA, principal coordinates analysis; PEth, phosphatidylethanol; Pielou, Pielou's evenness index; PIENTER‐3, Survey of Immunization Effect in the Netherlands for Evaluation of the National Immunization Program (third iteration); PLCO, prostate, lung, colorectal, and ovarian cancer screening trial; PLWH, people living with HIV; PO, prescription opioids; qPCR, quantitative polymerase chain reaction; SAH, severe alcohol‐associated hepatitis; Shannon, Shannon index; Simpson, Simpson index; Sobs, observed species richness; spp., species (plural, unspecified); STI, sexually transmitted infections; T1, timepoint 1 (at admission); T2, timepoint 2 (after withdrawal treatment); TLFB, timeline followback; UniFrac, weighted/unweighted UniFrac distance; VHD, very heavy drinkers.

#### 3.1.1 Study Designs and Populations

The majority of studies (*n* = 63) employed cross‐sectional designs, while 12 provided longitudinal data to assess microbiome dynamics over time. Most investigations focused on individuals with SUDs or heavy/harmful substance use, with several cohorts defined by clinical diagnostic criteria (e.g., DSM‐IV/DSM‐5 or ICD‐10) and others based on self‐reported frequency or intensity of use. Specifically, 47 studies included participants with clinically defined SUDs or heavy use, 9 examined frequency‐based substance use without formal SUD classification, and 19 reported substance use in a binary (yes/no) manner. Participant numbers varied widely—from fewer than 10 individuals per group to large‐scale studies involving thousands of subjects, such as those from the American Gut Project and multiethnic GWAS datasets. The majority of studies (63%) investigated microbiome associations with alcohol use. We found no studies examining the gut or oral microbiome in relation to MDMA or new psychoactive substances, such as synthetic cathinones or synthetic cannabinoids.

While most studies (*n* = 58) included both male and female participants, some targeted specific subpopulations (e.g., men who have sex with men (Kosnicki et al. 2019; Vujkovic‐Cvijin et al. 2020; Szóstak et al. 2023; Wang, Pan, et al. 2024; Fulcher et al. 2018; Cook et al. 2019; Nguyen et al. 2023), pregnant women and their newborns (Wang et al. 2021), or sexual and gender minorities (Morgan et al. 2024)). Certain studies featured specialized cohorts (e.g., intensive care unit (ICU) patients (Pettigrew et al. 2019)), which can complicate direct comparisons, but they were included given the scarcity of research on substance use–microbiome associations.

Of the studies included in this scoping review, 52% (*n* = 39) were rated as high quality (scoring ≥ 7) on the Newcastle‐Ottawa Scale or its adapted version, or using inherently low‐bias designs such as Mendelian randomization, whereas 43% (*n* = 32) were of moderate quality (scores 4–6), indicating higher risk of bias. Quality ratings were used descriptively and did not affect study inclusion.

Methodological heterogeneity and confounder assessment across studies are summarized in Table S1. Most studies excluded or adjusted for recent antibiotic or probiotic use; however, diet, socioeconomic status, medications, and major comorbidities were inconsistently addressed. Polysubstance use, a prevalent confounder in SUDs known to influence microbiome composition, was explicitly assessed in only a minority of studies, and adjustments were rare, highlighting a significant limitation and cautioning interpretation.

#### 3.1.2 Microbial Targets, Sampling Methods, and Sequencing Approaches

A diverse range of microbial targets was investigated. Specifically, 59 studies focused on the gut microbiome, 15 examined the oral microbiome, and 1 evaluated both. In addition, 4 studies addressed the gut mycobiome (Yang et al. 2017; Lang et al. 2020; Szóstak et al. 2023; Wang, Pan, et al. 2024) and 2 examined the gut virome (Jiang et al. 2020; Hsu et al. 2022). Most gut studies used fecal samples, though a few employed colonic or duodenal biopsies or rectal swabs. Oral microbiome studies typically collected saliva, with some using oral swabs or dental plaque.

For bacterial communities, 16S rRNA gene sequencing was the predominant approach (43 of the relevant studies), targeting various hypervariable regions (V1–V2, V3–V4, V4, V3–V5, V6–V8). Most used operational taxonomic units (OTUs) for taxonomic identification, though a subset employed amplicon sequence variants (ASVs) or oligotyping. A few studies used (Dubinkina et al. 2017; Kwan et al. 2022; Carbia et al. 2023; Wang, Pan, et al. 2024; Ward et al. 2023) shotgun metagenomic sequencing, enabling higher resolution analysis, identification at the species level, and more detailed taxonomic classification. Fungal studies primarily relied on ITS1 to characterize the mycobiome. Viral studies (Jiang et al. 2020; Hsu et al. 2022) used specialized pipelines to detect shifts in bacteriophage populations.

#### 3.1.3 Diversity Metrics and Abundance Analyses

A variety of metrics were used to assess alpha diversity, including the Shannon index, Chao1 index, Simpson index, ACE (abundance‐based coverage estimator) index, observed OTUs, Faith's phylogenetic diversity, and Pielou's evenness. Among these, Shannon and Chao1 were most frequent. About 25 studies reported no significant alpha‐diversity differences (16 alcohol, 2 cannabis, 1 cocaine, 6 methamphetamine [MA], 1 opioid), 10 observed increases (mostly in alcohol studies, plus 1 cannabis study), and 22 reported decreases (10 alcohol, 2 cannabis, 1 cocaine, 6 MA, 3 opioid).

Beta diversity was measured using weighted/unweighted UniFrac, Bray–Curtis, Jaccard, Euclidean, and Aitchison distances. Weighted UniFrac, Unweighted UniFrac, and Bray–Curtis were the most common. Although 15 studies reported no significant clustering, the majority (*n* = 37) found significant differences between substance users and controls.

Together, these findings offer a broad overview of the varied populations, methods, and microbial analyses used to explore the association between psychoactive substance use and microbiome composition. Detailed, substance‐specific results are presented in the following sections.

Given that gut‐bacterial analyses make up the majority of studies across all substance classes, Table 2 presents a concise cross‐substance overview of the percentage of studies reporting α‐diversity increases (↑), decreases (↓), or no change (↔), together with the proportion showing significant versus nonsignificant β‐diversity clustering.

**TABLE 2:** Cross‐substance comparison of α‐ and β‐diversity metrics in gut microbiome studies.

Substance	α‐Diversity			β‐Diversity	
	↓ (x/*N* %)	↑ (x/*N* %)	↔ (x/*N* %)	Clustering (x/*N* %)	No clustering (x/*N* %)
Alcohol	11/37 (30%)	10/37 (27%)	16/37 (43%)	22/31 (71%)	9/31 (29%)
Stimulants	4/10 (40%)	0/10 (0%)	6/10 (60%)	7/10 (70%)	3/10 (30%)
Opioids	3/4 (75%)	0/4 (0%)	1/4 (25%)	5/6 (83%)	1/6 (17%)
Cannabis	1/1 (100%)	0/1 (0%)	0/1 (0%)	0/1 (0%)	1/1 (100%)

*Note:* *N* = number of studies examining that diversity metric per substance. ↓ = reported decrease; ↑ = reported increase; ↔ = no change. “Clustering” = significant user‐vs‐control separation; “No clustering” = nonsignificant.

### 3.2 Microbiome Alterations Associated With Alcohol Use

A total of 48 studies examined gut or oral microbiome alterations related to alcohol use. Participant numbers ranged from very small cohorts (e.g., *n* = 6 (Zhao et al. 2020)) to large‐scale datasets, including the American Gut Project (Kosnicki et al. 2019) (*n* = 3447) and a multicohort genome‐wide association study (GWAS) with 18,340 participants (Wang, Pan, et al. 2024). Of these studies, 39 focused on the gut microbiome, 8 on the oral microbiome, and 1 analyzed both.

Most (*n* = 42) investigating bacterial communities relied on 16S rRNA gene sequencing, whereas a smaller subset (Tsuruya et al. 2016; Kim et al. 2020; Hsu et al. 2022; Hoang et al. 2023; Hoisington et al. 2024; Fan et al. 2018) employed shotgun metagenomic approaches. Three studies specifically addressed fungal components (Leclercq et al. 2014; Liao et al. 2018; Hoang et al. 2023), and two explored the virome (Maccioni et al. 2020; Wang et al. 2021); one study (Kyaw et al. 2023) evaluated both bacteria and fungi. Table 3 summarizes taxonomic shifts reported by each investigation, which varied from phylum‐to‐species analyses (Carbia et al. 2023) to focused examinations at the family or genus level (Rodríguez‐Rabassa et al. 2020; Hoisington et al. 2024) or even single‐species studies (Wang, Pan, et al. 2024; Ward et al. 2023). This methodological diversity, combined with differences in study design, sample type, and cohort size, reflects the overall heterogeneity of alcohol‐related microbiome research.

**TABLE 3:** Summary of gut and oral microbiome alterations in alcohol use‐related studies.

First author	Publication year	Gut or Oral?	Biome	Phylum ↑ Phylum ↓	Class ↑ Class ↓	Order ↑ Order ↓	Family ↑ Family ↓	Genus ↑ Genus ↓	Species ↑ Species ↓
Mutlu et al. (2012)	2012	Gut	Bacteria	—	—	—	↑ — *↓Bacteroidaceae*	—	—
Kakiyama et al. (2014)	2014	Gut	Bacteria	—	—	—	*↑Veillonellaceae* *↓Bacteroidaceae*	—	—
Leclercq et al. (2014)	2014	Gut	Bacteria	—	—	—	T1 vs. T2 in all AD subjects: ↑Erysipelotrichaceae↓ —	T1 vs. T2 in all AD subjects: ↑Holdemania↓ —	T1 vs. T2 in all AD subjects: —
							T1 vs. T2 in subjects with high intestinal permeability: ↑ — ↓*Ruminococcaceae*	T1 vs. T2 in subjects with high intestinal permeability: —	T1 vs. T2 in subjects with high intestinal permeability: ↑ — *↓Bifidobacterium* spp. and *Lactobacillus* spp.
Tsuruya et al. (2016)	2016	Gut	Bacteria	↑ — ↓*Bacteroidetes*	—	—	↑Streptococcaceae↓ —	*↑Streptococcus* *↓Bacteroides*, *Eubacterium*, *Anaerostipes*	—
Yang et al. (2017)	2017	Gut	Fungi	—	—	—	—	*↑Candida* *↓Epicoccum*, *unclassified fungi*, *Galactomyces*, and *Debaryomyces*	↑Candida dubliniensis↓ —
Dubinkina et al. (2017)	2017	Gut	Bacteria	—	—	—	—	ADS vs. Controls: *↑Klebsiella*, *Lactococcus* *↓Akkermansia*, *Coprococcus*, *unclassified Clostridiales*	ADS vs. Controls: *↑K. pneumoniae*, *Lactobacillus salivarius* , *Citrobacter koseri* , *Lactococcus lactis* subsp. *Cremoris* *↓Alistipes putredinis*, *Coprococcus eutactus* , *Ruminococcus lactaris* , *Streptococcus infantarius*
Liao et al. (2018)	2018	Gut	Bacteria	—	—	—	—	↑Bacteroides, Prevotella↓ —	—
Kosnicki et al. (2019)	2019	Gut	Bacteria	—	—	↑Clostridiales↓ —	*↑Barnesiellaceae, Ruminococcaceae, Coriobacteriaceae, RF32, S24‐7* *↓Clostridiaceae*	*↑Parabacteroides*, *Adlercreutzia*, *Anaerostipes*, *Oxalobacter*, *Paraprevotella*, *Phascolarctobacterium*, *Sutterella*, *Butyricimonas*, *YS2*, *Lachnobacterium*, *Odoribacter* *↓Peptococcus*	—
Lang et al. (2020)	2020	Gut	Fungi	↑Ascomycota↓ —	*↑Saccharomycetes* ↓*Eurotiomycetes*	*↑Saccharomycetales* ↓*Eurotiales*	*↑Saccharomycetaceae* *↓Trichocomaceae*	*↑Candida* *↓Penicillium*	—
Zhao et al. (2020)	2020	Gut	Bacteria	*↑Firmicutes*, *Tenericutes* ↓*Bacteroidetes*	↑Mollicutes, Gammaproteobacteria↓ —	↑Enterobacteriales↓ —	↑Enterobacteriaceae, Pasteurellaceae↓ —	*↑Pseudoramibacter_Eubacterium*, *Peptoniphilus*, *Lactococcus* *↓Eggerthella*, *Finegoldia*, *Anaerococcus*, *Anaerostipes*	—
Bjørkhaug et al. (2019)	2019	Gut	Bacteria	↑Proteobacteria↓ —	—	—	—	*↑Sutterella*, *Clostridium*, and *Holdemania* *↓Faecalibacterium*	—
Seo et al. (2020)	2020	Gut	Bacteria	—	—	—	—	AUDIT Zone III: *↑Prevotella* *↓Roseburia*	AUDIT Zone III: ↑Prevotella copri↓ —
Addolorato et al. (2020)*	2020	Gut	Bacteria	—	—	—	*↑Lachnospiraceae*, *Streptococcaceae*, *Enterobacteriaceae*, *Prevotellaceae*, *Staphylococcaceae*, *Lactobacillaceae* *↓Verrucomicrobiaceae*, *Bifidobacteriaceae*	*↑Sutterella*, *Streptococcus*, *Bacteroides*, *Prevotella*, *Lactobacillus*, *Staphylococcus* *↓Akkermansia*, *Bifidobacterium*, *Ruminococcus*	—
Rodríguez‐Rabassa et al. (2020)	2020	Oral	Bacteria	—	—	—	—	↑Prevotella↓ —	—
Smirnova et al. (2020)*	2020	Gut	Bacteria	*↑Firmicutes* ↓*Bacteroidetes* (HDC vs. HC)	—	—	*↑Enterobacteriaceae*, *Lactobacillaceae*, *Prevotellaceae*, *Streptococcaceae* *↓Acidaminococcaceae*, *Bacteroidaceae*, *Erysipelotrichaceae*, *Lachnospiraceae*	*↑Clostridium XIVa* and *Eisenbergiella*, *Prevotella* *↓*multiple genera from *Ruminococcaceae* and *Lachnospiraceae*	—
Adams et al. (2020)	2020	Gut	Bacteria	—	—	—	*↑Bacteroidales; other* *↓Lachnospiraceae*	—	—
Maccioni et al. (2020)	2020	Gut	Bacteria	—	—	—	—	Duodenum: ↑*Nubsella*, *Rothia*, and *Streptococcus* *↓Mycobacterium*, *Alcaligenes*, *Lachnoclostridium*, *Ralstonia*, *Rarobacter*, *Ethanoligenens*, and *Dolosigranulum*	—
								Gut: ↑Gemella, Actinomyces, Desulfovibrio, Subdoligranulum, and Akkermansia↓ —	
Ames et al. (2020)	2020	Gut	Bacteria	—	—	—	↑ — *↓Erysipelotrichaceae* and *Lachnospiraceae* (LHD > VHD)	—	—
Jiang et al. (2020)	2020	Gut	Viruses	—	—	—	↑Parvoviridae↓ —	↑Lactococcus phages↓ —	—
Maffei et al. (2021)	2021	Gut	Bacteria	—	—	—	↑Prevotellaceae (positively associated with PEth)↓ —	—	—
Lin et al. (2020)	2020	Gut	Bacteria	—	—	—	—	*↑Bacteroides* *↓Phascolarctobacterium, Ruminococcaceae_UCG‐ 002, Ruminococcaceae_UCG‐003, Ruminiclostridium_9*	—
González‐Zancada et al. (2020)	2020	Gut	Bacteria	—	—	—	↑ — *↓Clostridiaceae*	*↑Blautia*, *Pseudobutyrivibrio* *↓Alkaliphilus* (only in men)	*↑Blautia coccoides*, *Pseudobutyrivibrio xylanivorans* , *Johnsonella ignava* , *Blautia producta* *↓Alkaliphilus peptidifermentans*, *Clostridium hiranonis*
Gurwara et al. (2020)*	2020	Gut	Bacteria	LD: —	—	—	LD: —	LD: ↑Akkermansia, Lachnoclostridium↓ —	—
				HD: *↑Proteobacteria* ↓*Verrucomicrobia*			HD: ↑ — *↓Verrucomicrobiaceae*	HD: ↑*Escherichia*, *Haemophilus*, *Lachnospiraceae (UncO8895)* ↓*Faecalibacterium*, *Subdoligranulum*, *Sutterella*, *Alistipes*, *Roseburia*	
Vujkovic‐Cvijin et al. (2020)	2020	Gut	Bacteria	—	—	—	—	↑Bifidobacterium↓ —	—
Kim et al. (2020)	2020	Gut	Bacteria	—	—	—	—	↑Fusobacterium (p = 0.088)↓ —	—
Wang et al. (2021)	2021	Gut	Bacteria	—	—	—	—	Maternal: ↑*Phascolarctobacterium*, *Blautia* *↓Faecalibacterium*	—
								Infants: ↑Megamonas↓ —	
Kwan et al. (2022)	2022	Gut	Bacteria	—	—	—	↑Prevotellaceae↓ —	—	↑Prevotella copri↓ —
Hsu et al. (2022)	2022	Gut	Viruses	—	—	—	—	AUD: ↑two bacteriophages targeted *Streptococcus* and two targeted *Lactococcus*	—
								Active drinkers AUD vs. control: *↓Propionibacterium* phages	
								Increased in control Subjects: *↑Propionibacterium*, *Enterobacteria*, *Salmonella*, *Lactobacillus*, *Cronobacter*, *Escherichia*, and *Leuconostoc* phages	
								Drinking < Abstinent: ↓*Propionibacterium*, *Lactobacillus*, *Lactococcus*, *Leuconostoc*, and *Streptococcus* phages	
Hoang et al. (2023)	2023	Gut	Bacteria	—	↑Gammaproteobacteria↓ —	↑Enterobacteriales, Campylobacteriales↓ —	↑Micrococcaceae, Enterobacteriaceae, Enterococcaceae↓ —	*↑Rothia*, *Citrobacter*, *Enterococcus* *↓rc4_4*	*↑Citrobacter* sp., *Enterococcus* sp. *↓rc4_4* sp.
Carbia et al. (2023)	2023	Gut	Bacteria	—	—	—	—	*↑Veillonella* *↓Alistipes*	*↑Veillonella dispar* *↓Alistipes indistinctus*, *Alistipes shahii*
Szóstak et al. (2023)	2023	Gut	Fungi	—	—	—	—	—	Ever vs. never: ↑S. cerevisiae↓ —
									Daily: ↑*Z. mrakii* and *A. fumigatus* *↓B. cinerea*, M. restricta, *Y. lipolytica*, *U. maydis* , *A. fumigatus* , and *S. graminicola*
Zhao et al. (2023)*	2023	Gut	Bacteria	*↑Fusobacteria*, *Tenericutes* ↓*Bacteroidetes*	*↑Fusobacteriia*, *Actinobacteria*, *Mollicutes* ↓*Bacteroidia*, *Lentisphaeria*, and *Synergistia*	↑Fusobacteriales, Rickettsiales, and Actinomycetales↓ —	*↑Fusobacteriaceae* *↓*Ruminococcaceae, Lachnospiraceae, Bacteroidaceae, Clostridiaceae	*↑Bacteroides*, *Megamonas*, *Parabacteroides*, *Dorea*, *Enterobacter*, *Rahnella*, *Actinomyces* *↓Roseburia, Dialister*, *Coprococcus*, *Succinivibrio*, *Butyricicoccus*	Species from associated genera
Kyaw et al. (2023)	2023	Gut	Bacteria	↑Bacteroidota, Thermoplasmatota and Proteobacteria↓ —	—	—	—	—	↑Clostridium innocuum↓ —
Philips et al. (2023)	2023	Gut	Bacteria	—	—	—	—	Binge drinkers vs. daily drinkers: ↑Paracoccus↓ —	—
Wang, Yan, et al. (2023)*	2023	Gut	Bacteria + Fungi	Bacteria: ↑Patescibacteria and Fusobacteriota↓ —	Bacteria: ↑Saccharimonadia↓ —	Bacteria: ↑*Saccharimonadales*, *Fusobacteriales* ↓*Oscillospirales*	Bacteria: ↑*Saccharimonadaceae*, *Lachnospiraceae*, *Fusobacteriaceae*, *Erysipelotrichaceae* *↓Ruminococcaceae*, *Enterobacteriaceae*	Bacteria: ↑*Fusobacterium* *↓Roseburia*, *Anaerostipes*, *Ruminococcus*, *Dorea*, *Fisucatenibacter*, *Faecalibacterium*	Bacteria: ↑ — ↓*TM7x*
				Fungi: —	Fungi	Fungi —	Fungi	Fungi: ↑*Saccharomyces* and *Kurtzmaniella* *↓Candida*, *Papiliotrema*, *Lodderomyces*, *Alternaria*, *Kodamaea*, *Sporidiobolus*	Fungi: —
Qiao et al. (2024)	2024	Gut	Bacteria	—	—	—	Low‐to‐moderate vs. non‐drinking: ↑*Clostridiaceae* and *Peptostreptococcaceae* *↓Pasteurellaceae* and *Xanthomonadaceae*	Low‐to‐moderate vs. non‐drinking: ↑*Megamonas*, *Coprobacillus*, *Pseudobutyrivibrio*, and *Clostridium‐sensu‐stricto* *↓Bacteroides*	—
Hoisington et al. (2024)	2024	Gut	Bacteria	High PEth vs. low PEth: ↑ *Firmicutes* ↓*Bacteroidota*, *Verrucomicrobiota*; higher *Firmicutes*/ *Bacteroidetes* ratio	—	—	—	High PEth vs. low PEth: ↑ ‐↓*Akkermansia*, *Bacteroides*	—
Piacentino et al. (2024)*	2024	Gut	Bacteria	—	—	—	—	CD vs. HC: ↑*Lachnospira* *↓Dorea*	—
								CD vs. AB: ↑*Victivallis*, *Akkermansia*, *Fusicatenibacter*, *Dorea* *↓Eisenbergiella*, *Streptococcus*	
								AB vs. HC: ↑*Streptococcus* *↓Fusicatenibacter*, *Roseburia*, *Lachnospira*	
Wang, Pan, et al. (2024)	2024	Gut	Bacteria	—	—	—	↑Porphyromonadaceae↓ —	↑ — *↓Eubacterium ventriosum* group	—
Li et al. (2024)	2024	Gut	Bacteria	—	—	—	—	*↑Bacteroides* and *Fusobacterium* *↓Prevotella*, *Faecalibacterium* and *Roseburia*	—
Börnigen et al. (2017)	2017	Oral	Bacteria	—	—	—	—	—	↑g__Capnocytophaga|s__unclassified|4 392 350↓ —
Fan et al. (2018)	2018	Oral	Bacteria	HD: ↑ — ↓ *Firmicutes*	HD: ↑Bacilli↓ —	HD: —	—	HD: ↑Streptococcus, Lachnoanaerobaculum, Actinomyces↓ —	HD: ↑A. graevenitzzi↓ —
				HD and MD: —	HD and MD: —	HD and MD: ↑*Lactobacillales* ↓ — (HD and MD)		HD and MD: ↑Leptotrichia, Cardiobacterium, Neisseria↓ —	HD and MD: —
Ortiz et al. (2022)	2022	Oral	Bacteria	—	—	—	—	↑ — *↓Neisseria*, *Streptococcus*	—
Ward et al. (2023)	2023	Oral	Bacteria	—	—	↑ — ↓*Lactobacillales*, *Bacillales*, and *Flavobacteriales*	—	—	—
Yadav et al. (2023)*	2023	Oral	Bacteria	—	—	—	—	*↑Prevotella*, *Fusobacterium*, *Veillonella*, *Actinomyces*, *Bacteroidales*, *Corynebacterium* *↓Rothia*, *Tannerella*, *Clostridium*, *Streptococcus*	—
Maley et al. (2024)	2024	Oral	Bacteria	—	—	—	—	—	↑ — *↓Selenomonas* sp.*_oral_taxon_133*
Odendaal et al. (2024)*	2024	Oral	Bacteria	—	—	—	—	↑Stomatobaculum, Megasphaera, Actinomyces, Prevotella, Veillonella, Bifidobacterium↓ —	—

*Note:* Studies denoted with an asterisk (*) omit minor taxa to enhance clarity and comparability. Additionally, fungal taxa are included where reported.

Abbreviations: AB, abstinent; ADS, alcohol dependence syndrome; AUD, alcohol use disorder; AUDIT, alcohol use disorders identification test; BDs, binge drinkers; CD, current drinkers; HC, healthy controls; HD, heavy drinkers; HDC, heavy drinking controls; LD, light drinkers; LHD, less heavy drinkers; MD, moderate drinkers; Peth, phosphatidylethanol; spp. species (plural, unspecified); T1, timepoint 1 (at admission); T2, timepoint 2 (after withdrawal treatment); VHD, very heavy drinkers.

#### 3.2.1 Alcohol‐Associated Diversity and Taxonomic Composition Changes in the Gut Microbiome

Among the 37 studies examining alpha diversity, the Shannon index was the most frequent measure (29 studies). Of these, 16 reported no significant alpha‐diversity changes, 11 noted decreases, and 10 observed increases. Beta diversity was commonly assessed via Weighted UniFrac (11 studies), Bray–Curtis (O'Connor et al. 2020), and unweighted UniFrac (Mattick et al. 2014). Out of 31 studies reporting beta‐diversity findings, 22 found significant clustering, indicating an association between alcohol use and distinct gut microbial community structures.

A recurring theme involves phylum‐level shifts in *Firmicutes* and *Bacteroidetes*. Multiple investigations described increased *Firmicutes* (Zhao et al. 2020; Smirnova et al. 2020; Hoisington et al. 2024) alongside decreased *Bacteroidetes* (Tsuruya et al. 2016; Zhao et al. 2020, 2023; Smirnova et al. 2020; Hoisington et al. 2024), pointing to a potential *Firmicutes*‐dominant state in some patients with AUD. In addition, *Proteobacteria* often increased (Bjørkhaug et al. 2019; Gurwara et al. 2020; Kyaw et al. 2023), and *Verrucomicrobia* appeared reduced in heavy drinking subgroups (Gurwara et al. 2020; Hoisington et al. 2024). Large cohorts (Kosnicki et al. 2019; Wang, Pan, et al. 2024) observed subtler phylum‐level changes, supporting the notion that bigger sample sizes reveal more stable patterns.

At the genus level, studies frequently reported increases in *Bacteroides* (Liao et al. 2018; Addolorato et al. 2020; Lin et al. 2020; Zhao et al. 2023; Li et al. 2024), *Prevotella* (Liao et al. 2018; Addolorato et al. 2020; Rodríguez‐Rabassa et al. 2020; Smirnova et al. 2020), and *Streptococcus* (Tsuruya et al. 2016; Addolorato et al. 2020; Maccioni et al. 2020; Piacentino et al. 2024). *Sutterella* frequently appeared higher (Kosnicki et al. 2019; Bjørkhaug et al. 2019; Addolorato et al. 2020), except in one study (Gurwara et al. 2020). Meanwhile, potentially “beneficial” genera such as *Faecalibacterium* (Bjørkhaug et al. 2019; Gurwara et al. 2020; Wang et al. 2021; Wang, Yan, et al. 2023; Li et al. 2024), *Roseburia* (Seo et al. 2020; Gurwara et al. 2020; Zhao et al. 2023; Wang, Yan, et al. 2023; Piacentino et al. 2024; Li et al. 2024), and *Coprococcus* (Dubinkina et al. 2017; Zhao et al. 2023) often declined, implying a possible reduction in short‐chain fatty acid (SCFA) production. However, several genera showed contradictory patterns: for example, *Akkermansia* rose in certain contexts (Maccioni et al. 2020; Gurwara et al. 2020; Piacentino et al. 2024) but fell in others (Dubinkina et al. 2017; Addolorato et al. 2020; Hoisington et al. 2024), and *Bifidobacterium* increased in one study (Vujkovic‐Cvijin et al. 2020) yet declined in another (Addolorato et al. 2020). Such discrepancies may stem from differences in patient subgroups (e.g., liver disease, recent withdrawal) or smaller sample sizes. Of note, Wang, Pan, et al. (2024) implicated *Eubacterium ventriosum* group and *Porphyromonadaceae* as protective factors against alcohol misuse.

These inconsistencies may arise from cohort size, clinical severity, and host factors. For example, Leclercq et al. (2014) found that participants with AUD and high intestinal permeability exhibited pronounced reductions in *Ruminococcaceae*, illustrating the importance of subgroup analyses. Overall, increased *Bacteroides*/*Prevotella* and decreased *Faecalibacterium*/*Roseburia* recur across multiple studies but are far from universal. Larger datasets (Maffei et al. 2021; Kwan et al. 2022) reinforce the idea that certain taxa (e.g., *Prevotellaceae*) reliably rise in AUD, whereas smaller or more clinically tailored work points to subtype‐ or time‐dependent variations. Consequently, caution is advised when inferring causal links between alcohol use and specific gut microbes.

#### 3.2.2 Alcohol‐Associated Diversity and Taxonomic Compositional Changes in Oral Microbiome

Fewer studies (*n* = 8) investigated associations between alcohol use and oral microbiota but provided complementary insights. Four reported increases in alpha diversity, whereas four found no differences. Among the five investigations assessing beta diversity, two noted significant clustering of participants who consume alcohol versus controls, whereas three identified no major group‐level distinctions.

The range of genus‐level shifts indicates no uniform pattern. For instance, Ward et al. (2023) noted decreases in *Lactobacillales*, *Bacillales*, and *Flavobacteriales* among those with heavy alcohol consumption, while Ortiz et al. (2022) found lower *Neisseria* and *Streptococcus*. Additionally, Fan et al. (2018) documented decreased *Firmicutes* in heavy drinkers, but increased *Bacilli*, along with varying effects on *Lactobacillales*, *Streptococcus*, *Lachnoanaerobaculum*, and *Neisseria*. A large‐scale study by Odendaal et al. (2024) (*n* > 3000) identified multiple genera (e.g., *Stomatobaculum*, *Megasphaera*, *Actinomyces*, *Prevotella*, *Veillonella*, *Bifidobacterium*) enriched in alcohol‐consuming participants. In a comparative analysis, Ames et al. (2020) noted that 10 of 93 oral genera in AUD patients overlapped with their gut microbiomes—contrasting with 4 of 56 in the Human Microbiome Project—potentially highlighting novel oral–gut connections in alcohol use. Collectively, these findings highlight marked variability, with some taxa expanding in certain studies yet declining in others.

#### 3.2.3 Longitudinal Changes and the Abstinence Impact on Microbiome

A subset of studies leveraged longitudinal designs or tracked patients during withdrawal to evaluate how the gut microbiome may evolve with reduced alcohol intake. Leclercq et al. (2014) individuals with alcohol dependence from admission (T1) through 19 days of withdrawal (T2). Although overall diversity measures remained unchanged, only those with high intestinal permeability showed marked declines in *Ruminococcaceae* (*Faecalibacterium*, *Subdoligranulum*) and expansions in *Lachnospiraceae* (*Dorea*, *Blautia*). Maccioni et al. (2020) similarly noted compositional changes tied to intestinal permeability over a 3‐week withdrawal treatment program; α‐diversity remained static, with increased evenness seen only in AUD patients with altered gut barrier function, while β‐diversity showed minimal changes.

Other studies highlight the complexity of abstinence outcomes. Ames et al. (2020) found that both “Less Heavy Drinking” and “Very Heavy Drinking” subgroups underwent microbial shifts over time, though individual responses varied widely. Piacentino et al. (2024) further documented that abstinent participants with AUD had the most distinct gut microbiomes compared with current users or healthy controls, complicating assumptions of straightforward “recovery.” Additionally, Philips et al. (2023) observed that specific bacterial lineages (e.g., *Pedobacter*) at therapy initiation predicted relapse risk over 12 months in severe alcohol‐associated hepatitis.

Overall, while limited improvements can appear soon after abstinence—particularly in beneficial taxa—complete restoration of the microbiome may not occur within just a few weeks, especially for individuals with greater liver impairment or elevated intestinal permeability.

#### 3.2.4 Alcohol‐Associated Fungal and Viral Changes in the Gut Microbiome

Although not the main focus of this review, some investigations addressed fungal alterations in relation to harmful alcohol use. Yang et al. (2017) and Lang et al. (2020) both reported an overrepresentation of *Candida* in patients with AUD. A large Polish Microbiome Map cohort ((Szóstak et al. 2023), *n* = 923) found *S. cerevisiae* enriched in individuals who had ever consumed alcohol, while certain fungi (e.g., *Z. mrakii*, *A. fumigatus* ) became more prevalent in daily drinkers and others (e.g., *B. cinerea* , *M. restricta*, *Y. lipolytica*, *U. maydis* , *A. fumigatus* , and *S. graminicola* ) decreased. Wang, Yan, et al. (2023) likewise described a broader set of fungal genera increasing (e.g., *Saccharomyces*, *Kurtzmaniella*) or decreasing (*Candida*, *Papiliotrema*, *Lodderomyces*, *Alternaria*, *Kodamaea*, *Sporidiobolus*) in individuals with AUD compared with controls. These findings suggest that mycobiome disruptions can accompany alcohol misuse, though the directions and magnitude vary by cohort.

Studies on the viral fraction are fewer. Jiang et al. (2020) observed increased *Parvoviridae* and *Lactococcus* phages in patients with alcoholic hepatitis or AUD, possibly indicating an overrepresentation of bacteriophages affecting lactococcal hosts. Similarly, Hsu et al. (2022) documented an expansion of phages targeting *Streptococcus* and *Lactococcus* in active‐drinking AUD cohorts, whereas phages against *Propionibacterium*, *Lactobacillus*, *Leuconostoc*, and other genera were lower than in controls but rebounded during abstinence. Taken together, these data imply that alcohol‐related shifts in bacterial populations may parallel corresponding alterations in phage communities, highlighting the potential for broader ecosystem changes within the gut.

### 3.3 Microbiome Alterations Associated With Cannabis Use

#### 3.3.1 Cannabis‐Associated Gut and Oral Microbiome Diversity Changes

Six studies investigated the microbiome in relation to cannabis use: three addressed the gut microbiome (Morgan et al. 2024; Panee et al. 2018; Fulcher et al. 2018), and three focused on the oral microbiome (Ortiz et al. 2022; Newman et al. 2019; Luo et al. 2021). All employed 16S rRNA gene sequencing, with sample sizes ranging from 39 to 134 participants, illustrating a comparatively smaller evidence base than alcohol‐focused research.

Of the five studies examining microbial diversity, one gut investigation (Morgan et al. 2024) and one oral study (Luo et al. 2021) observed decreased alpha diversity in cannabis users. The remaining studies did not observe any differences. Beta‐diversity findings were mixed: one oral (Luo et al. 2021) and one gut study (Fulcher et al. 2018) documented distinct clustering between cannabis users and controls, whereas the others (Ortiz et al. 2022; Morgan et al. 2024; Newman et al. 2019) did not detect notable group‐level differences.

#### 3.3.2 Cannabis‐Associated Gut and Oral Microbiome Taxonomic Compositional Changes

Only four of the six cannabis‐focused investigations provided taxonomic data, and their diverse sampling sites (gut vs. oral) complicate any consistent overall trend (Table 4). For instance, *Streptococcus* decreased in the oropharynx of participants who smoke marijuana (Newman et al. 2019), yet increased in the saliva of cannabis users in another study (Luo et al. 2021). Similarly, *Fusobacteria* diminished at the tongue's lateral border in marijuana smokers (Newman et al. 2019) but increased in the gut of MSM living with HIV who also used marijuana (Fulcher et al. 2018). In gut‐focused studies, Panee et al. found elevated *Bacteroides* and lowered *Prevotella* (Panee et al. 2018), whereas Fulcher et al. noted higher *Fusobacterium* and *Anaerotruncus* alongside a decrease in *Dorea* (Fulcher et al. 2018). Meanwhile, Luo et al. (2021) reported oral dysbiosis characterized by surges in *Streptococcus* and *Actinomyces* and a reduction in *Neisseria* in patients with cannabis use disorder. Additionally, Newman et al. (2019) documented notably low levels of *Capnocytophaga*, *Fusobacterium*, and *Porphyromonas* on the tongue of marijuana users—taxa often enriched in head and neck cancers—and higher *Rothia*, which is usually suppressed in malignant mucosa. At the oropharynx site, *Selenomonas* was more abundant, but *Streptococcus* was reduced. Collectively, these findings demonstrate heterogeneity in cannabis‐related microbiome shifts across oral and gut sites. Given the limited number of studies and their varying designs, it remains challenging to define a stable pattern of cannabis‐associated taxonomic alterations.

**TABLE 4:** Summary of gut and oral microbiome alterations in cannabis use‐related studies.

First author	Publication year	Gut or Oral?	Phylum ↑ Phylum ↓	Family ↑ Family ↓	Genus ↑ Genus ↓	Species ↑ Species ↓
Panee et al. (2018)	2018	Gut	—	—	*↑Bacteroides* *↓Prevotella*	—
Fulcher et al. (2018)	2018	Gut	—	—	*↑Fusobacterium*, *Anaerotruncus* *↓Dorea*	—
Newman et al. (2019)	2019	Oral	—	—	Lateral border of the tongue: ↑*Rothia* and *Lautropia* *↓Fusobacterium*, *Porphyromonas*, and *Capnocytophaga*	Lateral border of the tongue: ↑Rothia mucilaginosa, Delftia acidovorans, Veillonella atypica and Bosea vestrisii↓—
					Oral pharynx: ↑*Veillonella*, *Mogobacterium* and *Selenomonas* *↓Streptococcus*	Oral pharynx: —
Luo et al. (2021)	2021	Oral	↑ — ↓*Proteobacteria*	—	*↑Actinomyces*, *Veillonella*, *Megasphaera*, *Streptococcus* *↓Neisseria*	*↑S* *. australis* , *S. bovis* , *S. gordonii* , *S. parasanguinis* , *S. uberis* , *A. lingnae*, *A. meyeri* , *A. odontolyticus* , *A. turicensis* *↓N* *. cinerea* , *N. elongata* , *N. flavescens* , *N. lactamica* , *N. mucosa* , *N. subflava*

### 3.4 Microbiome Alterations Associated With Stimulant Use

A total of 14 studies investigated the microbiome in relation to stimulant use, primarily focusing on methamphetamine (MA) (*n* = 9; (Fulcher et al. 2018; Cook et al. 2019; Yang, Yu, Liu, et al. 2021; Deng et al. 2021; Wang, Wang, et al. 2023; He et al. 2023; Liu et al. 2023; Liu et al. 2024; Deng et al. 2024; Yang, Yu, Yang, et al. 2021; Deng et al. 2022; Wang, Feng, et al. 2024)) and cocaine (*n* = 2; (Martinez et al. 2022; Fu et al. 2022)), with one study examining both MA and cannabis (Fulcher et al. 2018). Most (*n* = 10) sampled the gut microbiome via stool or rectal swabs (Martinez et al. 2022; Fulcher et al. 2018; Cook et al. 2019; Yang, Yu, Liu, et al. 2021; Deng et al. 2021, 2024; Wang, Wang, et al. 2023; He et al. 2023; Liu et al. 2023, 2024), whereas a smaller subset (*n* = 4) analyzed the oral microbiome from saliva or dental plaque (Fu et al. 2022; Yang, Yu, Yang, et al. 2021; Deng et al. 2022; Wang, Feng, et al. 2024). All employed 16S rRNA gene sequencing for bacterial profiling, with sample sizes ranging from 18 to 383 participants. Table 5 provides details on taxonomic changes.

**TABLE 5:** Summary of gut and oral microbiome alterations in stimulant use‐related studies.

First author	Publication year	Substance	Gut or Oral?	Phylum ↑ Phylum ↓	Class ↑ Class ↓	Order ↑ Order ↓	Family ↑ Family ↓	Genus ↑ Genus ↓	Species ↑ Species ↓
Martinez et al. (2022)	2022	Cocaine	Gut	↑ — ↓*Euryarchaeota*	—	—	↑ — ↓*Enterobacteriaceae*, *Muribaculaceae*	↑Lachnospira, Oscillospira↓ —	↑Bifidobacterium adolescentis↓ —
Fu et al. (2022)	2022	Cocaine	Oral	*↑Firmicutes* ↓*Proteobacteria*				*↑Streptococcus* *↓Actinobacillus*, *Campylobacter*, *Fusobacterium*, *Haemophilus*, *Mannheimia*, *Neisseria*, *Porphyromonas*	↑some *Streptococcus*‐related species ↓some *Neisseria*‐related species
Fulcher et al. (2018)	2018	MA	Gut	—	—	—	—	*↑Fusobacterium*, *Granulicatella*, and *Anaerococcus* *↓Parabacteroides*, *Collinsella*, *Paraprevotella*, *Fusicatenibacter*, *Blautia*, *Ruminococcus*, *Clostridium complex IV*, *Anaerotruncus*	—
Cook et al. (2019)	2019	MA	Gut	—	—	—	—	*↑Finegoldia*, *Fusobacterium*, *Parvimonas*, *Peptoniphilus*, *Peptostreptococcus*, *Porphyromonas* *↓Butyricicoccus*, *Faecalibacterium*	—
Yang, Yu, Liu, et al. (2021)	2021	MA	Gut	—	—	*↑Clostridiales*, *Sphingomonadales, Xanthomonadales* ↓*Desulfovibrionales*	*↑Sphingomonadales*, *Xanthomonadales*, *Romboutsia*, *Lachnospiraceae* *↓Deltaproteobacteria*, *Bacteroidaceae*	Low abundances	—
Deng et al. 2021	2021	MA	Gut	↑Actinobacteria↓ —	*↑Betaproteobacteria*, *Actinobacteria* ↓*Bacilli*	*↑Burkholderiales* and *Coriobacteriales* ↓*Lactobacillales*	*↑Coriobacteriaceae* *↓Streptococcaceae*	*↑Megasphaera*, *Conllinsella*, *Odoribacter* *↓Faecalibacterium*, *Blautia*, *Dorea*, *Streptococcus*	—
Wang, Wang, et al. (2023)	2023	MA	Gut	↑Proteobacteria↓ —	—	—	*↑Bifidobacteriaceae*, *Enterobacteriaceae* *↓Ruminococcaceae*	*↑Escherichia–Shigella* *↓Faecalibacterium*	—
He et al. (2023)	2023	MA	Gut	—	↑Alphaproteobacteria↓ —	↑Oceanospirillales, Xanthomonadales, Rhizobiales↓ —	↑Clostridiaceae, Halomonadaceae, Hyphomicrobiaceae, Xanthomonadaceae↓ —	↑Halomonas, Clostridium, Devosia, Dorea↓ —	—
Liu et al. (2023)	2023	MA	Gut	*↑Firmicutes*, *Proteobacteria* higher *Firmicutes*/*Bacteroidetes* ratio ↓*Actinobacteria*	—	—	—	*↑Lachnospira*, *Lachnoclostridium* *↓Bifidobacterium*	—
Liu et al. (2024)	2024	MA	Gut	MA vs. HC: ↑*Firmicutes* and *Synergistetes* ↓*Actinobacteria* and *Bacteroidetes*	MA vs. HC: ↑Bacilli, *Synergistia*, and *Clostridia* ↓*Actinobacteria*, *Bacteroidia*, and *Negativicutes*	MA vs. HC: *↑Actinomycetales*, *Micrococcales*, *Lactobacillales*, *Clostridiales*, *Aeromonadales*, and *Synergistales* ↓*Bifidobacteriales*, *Bacteroidales*, and *Selenomonadales*	MA vs. HC: *↑Actinomycetaceae*, *Lachnospiraceae*, *Aeromonadaceae*, *Succinivibrionaceae*, and *Synergistaceae* *↓Bifidobacteriaceae*, *Bacteroidaceae*, *Porphyromonadaceae*, *Rikenellaceae*, *Enterococcaceae*, and *Leuconostocaceae*	MA vs. HC: ↑*Escherichia–Shigella*, *Lachnoclostridium, Sutterella, Alloprevotella, Tyzzerella_4*, *Faecalitalea*, *Eisenbergiella*, *Succinivibrio*, *Enterobacter*, *Actinomyces*, *Peptostreptococcus*, and *Citrobacter* ↓Bifidobacterium, *Bacteroides*, *Butyricimonas*, *Odoribacter*, *Parabacteroides*, *Prevotellaceae_NK3B31_group*, *Alistipes*, *Enterococcus*, *Weissella*, *Lactococcus*, *Coprococcus_3*, *Lachnospiraceae_FCS020_group*, *Lachnospiraceae_ND3007_group*, *Ruminiclostridium*, *Ruminococcaceae_UCG_003*, *Ruminococcaceae_UCG_013*, *Erysipelotrichaceae_UCG_003*, and *Parasutterella*	—
				MA Baseline vs. MA Follow‐up: ↑Firmicutes↓ —	MA Baseline vs. MA Follow‐up: ↑Clostridia↓ —	MA Baseline vs. MA Follow‐up: ↑Clostridiales and Rhodobacterales↓ —	MA Baseline vs. MA Follow‐up: ↑*Rhodobacteraceae* *↓Lachnospiraceae*	MA Baseline vs. MA Follow‐up: ↑*Eisenbergiella*, *Candidatus_Soleaferrea*, *Paracoccus*, and *Citrobacter* *↓Gardnerella*, *Prevotella_7*, *Howardella*, *Catenibacterium*, and *Faecalitalea*	
Deng et al. (2024)	2024	MA	Gut	MA‐GS vs. HC: ↑*Firmicutes* ↓*Actinobacteria*, *Bacteroidetes*	MA‐GS vs. HC: ↑ — ↓*Actinobacteria*	MA‐GS vs. HC: ↑*Micrococcales*, *Aeromonadales* ↓*Bifidobacteriales*	MA‐GS vs. HC: ↑*Micrococcaceae* *↓Bifidobacteriaceae*	MA‐GS vs. HC: ↑*Faecalitalea* *↓Bifidobacterium*, *Weissella*	—
Yang, Yu, Yang, et al. (2021)	2021	MA	Oral	MUD vs. HC: —	MUD vs. HC: ↑Negativicutes↓ —	MUD vs. HC: —	MUD vs. HC: ↑*Veillonellaceae* and *Cryptosporangiaceae* *↓*undefined *Spirochaetes* and *Thermomonosporaceae*	MUD vs. HC: ↑Veillonella↓ —	MUD vs. HC: —
				MUD after 2 weeks: —	MUD after 2 weeks: —	MUD after 2 weeks: —	MUD after 2 weeks: —	MUD after 2 weeks: —	MUD after 2 weeks: —
Deng et al. (2022)	2022	MA	Oral	*↑Proteobacteria*, *Bacteroidetes* ↓*Firmicutes*, *Actinobacteria*, *Fusobacteria*	—	—	—	*↑Neisseria*, *Porphyromonas*, *Prevotella*, *Fusobacterium*, *Haemophilus* *↓Veillonella*, *Streptococcus*, *Leptotrichia*, *Actinomyces*	—
Wang, Feng, et al. (2024)	2024	MA	Oral	—	—	—	—	*↑Granulicatella*, *Gemella*, and *Peptostreptococcus*, *Parvimonas*, *unclassified_Leptotrichiaceae*, *Abiotrophia* *↓Campylobacter*, *Aggregatibacter*, *Dialister*, *Corynebacterium*, *Alloprevotella*, *Bergeyella*, *Filifactor*, *Fusobacterium*, *Lautropia*, *Megasphaera*, *unclasiified_Clostridiales_bacterium_oral_taxon_075*	—

Abbreviations: HC, healthy controls; MA, methamphetamine; MA‐GS, methamphetamine—good sleep group; MUD, methamphetamine use disorder.

#### 3.4.1 Stimulant‐Associated Diversity and Taxonomic Composition Changes in the Gut Microbiome

Among the 10 gut‐focused studies on stimulant use, seven detected significant clustering between participants who use stimulants and controls in beta‐diversity analyses (e.g., Bray–Curtis, Weighted UniFrac), indicating distinct microbial community structures. Regarding alpha diversity, four studies reported decreases in individuals who use stimulants (Yang, Yu, Liu, et al. 2021; Wang, Wang, et al. 2023; Liu et al. 2023, 2024), whereas none observed an increase.

At the phylum level, multiple investigations noted elevated *Firmicutes* (Liu et al. 2023, 2024; Deng et al. 2024) and lower *Bacteroidetes* (Liu et al. 2024; Deng et al. 2024) in participants who use MA, resulting in an increased *Firmicutes*/*Bacteroidetes* ratio indicating gut dysbiosis. Two studies documented elevated *Proteobacteria* (Wang, Wang, et al. 2023; Liu et al. 2023) while three noted a reduction in *Actinobacteria* (Liu et al. 2023, 2024; Deng et al. 2024).

Additional insights emerged at the family and genus levels. In two studies (Yang, Yu, Liu, et al. 2021; Liu et al. 2024), the family *Lachnospiraceae* was higher among participants who use stimulants, whereas generally *Faecalibacterium* (Cook et al. 2019; Deng et al. 2021; Wang, Wang, et al. 2023) and *Bifidobacterium* (Liu et al. 2023, 2024; Deng et al. 2024) decreased. However, some results were inconsistent: *Bifidobacteriaceae* levels dropped in two investigations (Liu et al. 2024; Deng et al. 2024) but increased in another (Wang, Wang, et al. 2023). The latter study found that this increase was exclusive to females with metamphetamine use disorder, suggesting that sex may influence the structure of the gut microbiota. In a separate observation, *Enterobacteriaceae* increased in a MA study (Wang, Wang, et al. 2023) but decreased in a cocaine study (Martinez et al. 2022), suggesting that comparisons may be influenced by the specific type of stimulant involved.

Yet, the overall results point to a potential trend of opportunistic or proinflammatory taxa (e.g., *Escherichia*–*Shigella* (Wang, Wang, et al. 2023; Liu et al. 2024)) expanding in association with stimulant use, while beneficial or SCFA‐producing genera (e.g., *Faecalibacterium* (Cook et al. 2019; Deng et al. 2021; Wang, Wang, et al. 2023), *Bifidobacterium* (Liu et al. 2023, 2024; Deng et al. 2024)) often decline.

#### 3.4.2 Stimulant‐Associated Diversity and Taxonomic Composition Changes in the Oral Microbiome

Four studies addressed oral microbial communities in relation to stimulant use (Fu et al. 2022; Yang, Yu, Yang, et al. 2021; Deng et al. 2022; Wang, Feng, et al. 2024). Three (Fu et al. 2022; Yang, Yu, Yang, et al. 2021; Wang, Feng, et al. 2024) observed lower alpha diversity in participants who use cocaine or MA, whereas one (Deng et al. 2022) identified no alpha‐diversity differences. Beta‐diversity analyses were assessed in three of the four oral microbiome studies (Yang, Yu, Yang, et al. 2021; Deng et al. 2022; Wang, Feng, et al. 2024), consistently showing significant clustering between participants and controls.

Reported taxonomic shifts in the oral microbiome varied. For example, Fu et al. (cocaine) observed increased *Firmicutes* and *Streptococcus* but decreased *Proteobacteria*, *Neisseria*, and *Fusobacterium* (Fu et al. 2022), whereas Deng et al. (MA) showed the opposite for these same taxa (Deng et al. 2022). Similarly, *Veillonella* rose in Yang, Yu, Yang, et al. (2021), but declined in Deng et al. (2022). Moreover, a larger study by Wang, Feng, et al. (2024) (*n* = 278 participants who use MA vs. 105 controls) found elevated *Granulicatella*, *Gemella*, *Peptostreptococcus*, *Parvimonas*, and others, whereas *Fusobacterium* declined—contradicting Deng et al. findings in a smaller (*n* = 45) MA study (Deng et al. 2022). Notably, the study by Deng et al. (2022) sampled dental plaque rather than saliva and included only female participants, which may partially account for the observed discrepancies compared with other studies. Although stimulant use is associated with distinct oral microbiome shifts—often reflected in alpha and beta diversity—the small sample sizes and methodological variability limit the ability to define a consistent microbial signature.

#### 3.4.3 Stimulants Abstinence Impact on Longitudinal Changes of the Gut Microbiome

Two studies employed longitudinal designs to assess how the microbiome evolves with reduced MA use. Yang, Yu, Yang, et al. (2021) followed 12 participants for 2 weeks of abstinence plus olanzapine therapy and noted no significant changes in alpha or beta diversity, nor in specific taxa, suggesting that 2 weeks may be insufficient to reverse MA‐related alterations in the oral microbiome. In contrast, Liu et al. (2024) tracked 25 individuals who ceased MA for 2 months and found minimal alpha‐diversity changes but significant beta‐diversity shifts between baseline, follow‐up, and control groups. Additional analyses (LefSe) indicated decreases in opportunistic taxa (e.g., *Enterococcus*) alongside increases in butyrate‐producing bacteria (e.g., *Acidaminococcus*) suggesting partial microbiome recovery over a longer abstinence period. Overall, these limited findings imply that while short‐term abstinence (2 weeks) yields negligible improvements, a longer cessation period (2 months) may promote more favorable compositional shifts, though returning fully to “normal” remains uncertain and warrants extended follow‐up.

### 3.5 Microbiome Alterations Associated With Opioid Use

Nine studies have examined the relationship between opioid use and the microbiome, with eight focusing on the gut microbiome (Acharya et al. 2017; Barengolts et al. 2018; Pettigrew et al. 2019; Li et al. 2020; Gicquelais et al. 2020; Cruz‐Lebrón et al. 2021; Nguyen et al. 2023; Xie et al. 2024) and one on the oral microbiome (Wu et al. 2021). All studies employed 16S rRNA gene sequencing, with one study also incorporating shotgun metagenomic sequencing (Nguyen et al. 2023). Sample sizes ranged from cohorts of 58 participants (Xie et al. 2024) to larger datasets of several hundred (Wu et al. 2021), with one study including 9167 fecal samples from 1201 cancer patients with documented opioid exposures (Nguyen et al. 2023).

#### 3.5.1 Opioid‐Associated Diversity Changes

Four studies examined alpha diversity (Li et al. 2020; Gicquelais et al. 2020; Cruz‐Lebrón et al. 2021; Wu et al. 2021), with three (Gicquelais et al. 2020; Cruz‐Lebrón et al. 2021; Wu et al. 2021) reporting a decrease associated with opioid use and one (Li et al. 2020) finding no significant difference. Gicquelais et al. (2020) also noted reduced alpha diversity in opioid agonist users compared with nonusers, though no significant differences emerged in those receiving combination treatments (agonist + antagonist) or solely antagonist therapy.

Beta diversity was evaluated in six studies (Acharya et al. 2017; Li et al. 2020; Gicquelais et al. 2020; Cruz‐Lebrón et al. 2021; Xie et al. 2024; Wu et al. 2021), with five reporting significant clustering between opioid users and control groups (Acharya et al. 2017; Li et al. 2020; Cruz‐Lebrón et al. 2021; Xie et al. 2024; Wu et al. 2021), suggesting distinct microbial community structures associated with opioid exposure. These findings collectively point to a common pattern of reduced alpha diversity and distinct beta diversity in opioid users, suggesting gut microbiome imbalance. However, the wide range of participant numbers, diverse clinical contexts (e.g., cirrhosis, ICU patients), and differing opioid use parameters complicate direct comparisons across studies.

#### 3.5.2 Opioid‐Associated Taxonomic Compositional Changes

Detailed taxonomic shifts linked to opioid use are summarized in Table 6. At the phylum level, three gut studies (Li et al. 2020; Cruz‐Lebrón et al. 2021; Xie et al. 2024) and one oral study (Wu et al. 2021) observed increased *Actinobacteria*. Several investigations also reported elevated *Bifidobacteriaceae* at the family level (Acharya et al. 2017; Cruz‐Lebrón et al. 2021; Xie et al. 2024), corresponding to higher *Bifidobacterium* in four studies (Acharya et al. 2017; Barengolts et al. 2018; Li et al. 2020; Xie et al. 2024). Other genera showed more variable patterns: *Roseburia* was reduced in two reports (Acharya et al. 2017; Gicquelais et al. 2020), but increased in one (Li et al. 2020) whereas *Lactobacillus* rose in two studies (Li et al. 2020; Gicquelais et al. 2020), yet declined in another (Pettigrew et al. 2019).

**TABLE 6:** Summary of gut and oral microbiome alterations in opioid use‐related studies.

First author	Publication year	Gut or Oral?	Phylum ↑ Phylum ↓	Class ↑ Class ↓	Order ↑ Order ↓	Family ↑ Family ↓	Genus ↑ Genus ↓	Species ↑ Species ↓
Acharya et al. (2017)	2017	Gut	—	HE Op: ↑ — ↓*Clostridia*	HE Op: ↑*Bifidobacteriales* ↓*Clostridiales*	HE Op: ↑*Bifidobacteriaceae* *↓Streptococcaceae, Lachnospiraceae, Bacteroidaceae, Clostridiales XIV*	HE Op: ↑*Bifidobacterium* *↓Roseburia*	—
				Non‐HE Op: —	Non‐HE Op: —	Non‐HE Op: ↑Peptostreptococcaceae↓ —	Non‐HE Op: ↑*Peptostreptococcaceae. Other* *↓Parasutterella*	
Barengolts et al. (2018)	2018	Gut	—	—	↑Lactobacillales↓ —	—	↑Bifidobacterium↓ —	—
Pettigrew et al. (2019)	2019	Gut	—	—	—	—	↑ — *↓Blautia* and *Lactobacillus*	—
Li et al. (2020)	2020	Gut	MP > CDet and HC: ↑Actinobacteria	—	—	—	MP: ↑*Lactobacillus*, *Streptococcus*, *Veillonella*, *Bifidobacterium*, *Intestinibacter*, *Klebsiella*, and *Fusicatenibacter*	
			DU > CDet: ↑*Firmicutes*				DU: ↑*Ruminococcus*, *Roseburia*, *Collinsella*, and *Succinivibrio*	
							*CDet:* *↑Alloprevotella*, *Erysipelotrichaceae incertae sedis*, and *Flavonifractor*	
							*HC:* ↑*Aestuariispira*	
Gicquelais et al. (2020)	2020	Gut	—	—	—	—	Ag vs. N: ↑*Unclassified Enterobacteriaceae*, *Lactobacillus*, *Clostridium cluster XIVa*, *Faecalicoccus*, *Anaerostipes*, *Streptococcus* ↓*Unclassified Firmicutes*, *Bilophila*, *Roseburia*	—
Cruz‐Lebrón et al. (2021)	2021	Gut	*↑Actinobacteria* ↓*VerrucomicrobiaI*	—	—	*↑Bifidobacteriaceae* *↓Akkermasiaceae*	—	*↑Bifidobacterium bifidum*, *Bifidobacterium longum* *↓Akkermansia muciniphila*
Nguyen et al. (2023)	2023	Gut	—	—	—	—	↑Enterococcus↓ —	—
Xie et al. (2024)	2024	Gut	↑Actinobacteria↓ —	—	—	*↑Bifidobacteriaceae*, *Lachnospiraceae* *↓Ruminococcaceae*	*↑Bifidobacterium* ↓*Faecalibacterium*, *Megamonas*	—
Wu et al. (2021)	2021	Oral	Opium‐only vs. Never cigarettes or opium: ↑Firmicutes↓ —	—	—	—	Opium‐only vs. Never cigarettes or opium: ↑ — *↓Abiotrophia*, *Lautropia*	—
			Opium and cigarettes vs. Never cigarettes or opium: ↑*Actinobacteria* ↓*Bacteroidetes*, *Proteobacteria*				Opium and cigarettes vs. Never cigarettes or opium: —	

Abbreviations: Ag, opioid agonist; AgAt, opioid agonist and antagonist; At, opioid antagonist; CDet, compulsory detention patients; DU, drug users; HC, healthy controls; HE Op, patients with hepatic encephalopathy on opioids; MP, methadone maintenance treatment patients; N, neither opioid agonist or antagonist; Non‐HE Op, patients without hepatic encephalopathy on opioids.

Further insights emerged from studies in specific clinical cohorts. In individuals with type 2 diabetes (T2D) not taking metformin, Barengolts et al. found a significant opioid‐related increase in *Bifidobacterium* (Barengolts et al. 2018). Among ICU patients, Pettigrew et al. observed lower *Blautia* and *Lactobacillus* in those receiving opioids compared with nonusers (Pettigrew et al. 2019). Meanwhile, Cruz‐Lebrón et al. (2021) reported that individuals on methadone had decreased *Akkermansia muciniphila* , a microbe that supports gut barrier integrity, and that long‐term methadone treatment (≥ 10 years) was linked to an increased *Bacteroidetes*/*Firmicutes* ratio. Additionally, Li et al. (2020) showed that methadone maintenance therapy (MMT) participants harbored greater abundances of multiple significantly different taxa, potentially reflecting elevated nutrient intake and orexin A levels that favor beneficial bacteria.

Taken together, these findings suggest a possible trend of increased *Bifidobacterium* in opioid‐exposed individuals, particularly those undergoing methadone treatment. However, variability across studies—exemplified by *Roseburia* and *Lactobacillus*—along with heterogeneity in clinical populations (e.g., OUD, T2D, ICU) necessitates cautious interpretation.

### 3.6 Cross‐Substance Comparative Taxonomic Overview

In gut‐bacterial studies, cross‐substance comparisons are necessarily limited by sparse overlap, but a few consistent shifts emerge. At the phylum level, *Firmicutes* increases were reported in three alcohol (Zhao et al. 2020; Smirnova et al. 2020; Hoisington et al. 2024), three stimulant (Liu et al. 2023, 2024; Deng et al. 2024), and one opioid (Wu et al. 2021) investigation; *Bacteroidetes* declines in five alcohol (Tsuruya et al. 2016; Zhao et al. 2020, 2023; Smirnova et al. 2020; Hoisington et al. 2024) and two stimulant (Liu et al. 2024; Deng et al. 2024) studies; *Proteobacteria* rises in three alcohol (Bjørkhaug et al. 2019; Gurwara et al. 2020; Kyaw et al. 2023) and two stimulant reports (Wang, Wang, et al. 2023; Liu et al. 2023); and *Verrucomicrobia* reductions in two alcohol (Gurwara et al. 2020; Hoisington et al. 2024) and one opioid cohort (Cruz‐Lebrón et al. 2021). At the genus level, the SCFA‐producer *Faecalibacterium* was depleted in five alcohol (Bjørkhaug et al. 2019; Gurwara et al. 2020; Wang et al. 2021; Wang, Yan, et al. 2023; Li et al. 2024), three stimulant (Cook et al. 2019; Deng et al. 2021; Wang, Wang, et al. 2023) and one opioid (Xie et al. 2024) studies—the most reproducible finding—while *Escherichia–Shigella* elevations recurred in one alcohol (Gurwara et al. 2020) and two stimulant (Wang, Wang, et al. 2023; Liu et al. 2024) investigations. The abundance of butyrate‐producing *Roseburia* was decreased in six alcohol studies (Seo et al. 2020; Gurwara et al. 2020; Zhao et al. 2023; Wang, Yan, et al. 2023; Piacentino et al. 2024; Li et al. 2024) whereas its changes were unreported in studies of cannabis and stimulants and were less consistent in opioid studies (Acharya et al. 2017; Li et al. 2020; Gicquelais et al. 2020). *Bifidobacterium* was reported as more abundant in four opioid studies (Acharya et al. 2017; Barengolts et al. 2018; Li et al. 2020; Xie et al. 2024) and less abundant in three stimulant reports (Liu et al. 2023, 2024; Deng et al. 2024). Other taxa (e.g., *Actinobacteria*, *Coprococcus*) showed mixed or substance‐specific directions, and cannabis studies remain too few for meaningful crossover analysis. Detailed findings are summarized in Table 7.

**TABLE 7:** Directional shifts of key gut microbiome taxa across substance use categories.

	Alcohol (*N* = 34)	Cannabis (*N* = 2)	Stimulants (*N* = 10)	Opioids (*N* = 8)
Firmicutes	3↑	—	3↑	1↑
Bacteroidetes	5↓1↑	—	2↓	—
Proteobacteria	3↑	—	2↑	—
Verrucomicrobia	2↓	—	—	1↓
Actinobacteria	1↑	—	3↓, 1↓	3↑
Lachnospiraceae	4↓, 2↑	—	2↑	1↑, 1↓
Bifidobacteriaceae	1↓	—	1↑ 2↓	3↑
Enterobacteriaceae	4↑, 1↓	—	1↑(MA), 1↓ (Coc)	—
Bacteroides	5↑, 2↓	1↑	1↓	—
*Prevotella*	4↑, 1↓	1↓	1↓	—
*Streptococcus*	4↑	—	1↓	2↑
*Sutterella*	3↑, 1↓	—	1↑	—
*Faecalibacterium*	5↓	—	3↓	1↓
*Roseburia*	6↓	—	—	2↓, 1↑
*Coprococcus*	2↓	—	—	—
*Akkermansia*	3↑, 3↓	—	—	—
*Bifidobacterium*	1↑, 1↓	—	3↓	4↑
*Lactobacillus*	2↑, 1↓	—	—	2↑, 1↓
*Fusobacterium*	3↑	1↑	2↓	—
*Anaerotruncus*	—	1 ↑	1↓	—
*Escherichia*–*Shigella*	1↑ (*Escherichia*)	—	2↑	—

*Note:* ↑ = number of studies reporting an increase; ↓ = number of studies reporting a decrease; — = taxon not assessed in that category.

Across oral‐bacterial studies (8 alcohol, 4 stimulants, 2 cannabis, 1 opioid), the evidence remains too sparse and heterogeneous—varying sample sites (tongue, saliva, plaque) and small cohorts—to justify a formal cross‐substance review. Instead, we note a couple of genera that recur across multiple substance classes despite these limitations: *Veillonella* was reported as enriched in alcohol (Yadav et al. 2023; Odendaal et al. 2024), stimulant (Yang, Yu, Yang, et al. 2021) and both cannabis studies (Newman et al. 2019; Luo et al. 2021), and *Actinomyces* appeared elevated in three alcohol (Fan et al. 2018; Yadav et al. 2023; Odendaal et al. 2024) and one cannabis investigation (Luo et al. 2021). All other taxa (e.g., *Streptococcus*, *Neisseria*, *Fusobacterium*) showed inconsistent directions between studies. Detailed, substance‐specific oral findings are described previously in Sections 3.2, 3.5.

## 4 Discussion

This review included 75 clinical studies linking gut or oral microbiomes to psychoactive substance use. While alcohol‐focused investigations dominated (48 studies), fewer examined stimulants (Nielsen et al. 2019), opioids (Mattick et al. 2014), and cannabis (Rösner et al. 2010). Alpha diversity, a key indicator of gut microbiota health, generally reflects greater stability and ecological function when higher, contributing to a more resilient microbiota. In this review, changes in alpha diversity appeared substance‐specific: stimulant and opioid use were often associated with declines, whereas findings related to alcohol were more variable. Most studies also reported compositional differences between substance users and controls, as reflected in beta diversity. Taxonomic changes, such as reduced *Faecalibacterium* or expanded *Bifidobacterium*, were repeatedly noted but strongly modulated by clinical context.

Alcohol remains the most extensively studied. While comparable numbers of investigations report no changes, decreases, or increases in alpha diversity, the majority show notable clustering in beta diversity, implying that overall community composition often differs between alcohol users and controls. Many studies suggest shifts toward elevated *Firmicutes* (Zhao et al. 2020; Smirnova et al. 2020; Hoisington et al. 2024) and *Proteobacteria* (Bjørkhaug et al. 2019; Gurwara et al. 2020; Kyaw et al. 2023) and reduced *Bacteroidetes* (Tsuruya et al. 2016; Zhao et al. 2020, 2023). Preclinical data on pathogen‐induced gut dysbiosis demonstrate that inflammation, epithelial injury, and reduced butyrate production increase oxygen availability and host‐derived electron acceptors such as nitrate. These changes disrupt the anaerobic environment of the colon and provide electron acceptors for respiration, creating conditions that favor the growth of facultative anaerobes—particularly *Gammaproteobacteria* (phylum *Proteobacteria*) and *Bacilli* (phylum *Firmicutes*)—over obligate fermenters, such as *Clostridia* (phylum *Firmicutes*) and *Bacteroidia* (phylum *Bacteroidetes*) (Winter and Bäumler 2023). Butyrate depletion impairs mitochondrial oxygen consumption in colonocytes, further raising epithelial oxygen levels and exacerbating dysbiosis (Rivera‐Chávez et al. 2016).

This altered microbial profile—characterized by increased *Proteobacteria* and *facultative Firmicutes* and decreased *Clostridia* and *Bacteroidia*—is also observed in various noncommunicable diseases such as inflammatory bowel disease (Shin et al. 2015; Litvak et al. 2017; Frank et al. 2007). The current review highlights this pattern across multiple studies, showing a consistent decrease in *Bacteroidetes* (Tsuruya et al. 2016; Zhao et al. 2020, 2023; Smirnova et al. 2020; Hoisington et al. 2024) and an enrichment of *Firmicutes* and *Proteobacteria* in individuals with alcohol use (Zhao et al. 2020; Bjørkhaug et al. 2019; Smirnova et al. 2020; Gurwara et al. 2020; Kyaw et al. 2023; Hoisington et al. 2024). At the genus level, *Bacteroides* and *Prevotella* often appeared increased—both of which have been tied to underlying dietary habits (Gorvitovskaia et al. 2016), with *Prevotella* commonly enriched in both the gut and oral microbiome (Liao et al. 2018; Seo et al. 2020; Addolorato et al. 2020; Rodríguez‐Rabassa et al. 2020; Smirnova et al. 2020; Yadav et al. 2023; Odendaal et al. 2024). Although *Prevotella* is traditionally viewed as commensal, certain strains may act as inflammophilic pathobionts, driving Th‐17 –mediated inflammation in a dysbiotic environment (Larsen 2017). The butyrate‐producing *Faecalibacterium, Coprococcus*, and *Roseburia* tend to decline (Parada Venegas et al. 2019; Faden 2022; Escalante et al. 2025). In contrast, the opportunistic, proinflammatory pathogens, such as *Enterobacteriaceae* (including *Escherichia, Klebsiella*), expand (Dubinkina et al. 2017; Zhao et al. 2020; Addolorato et al. 2020; Smirnova et al. 2020; Hoang et al. 2023), indicating that the gut microbiota tilts toward dysbiosis and exacerbates inflammation in alcohol use.

Nonetheless, contradictory findings for *Akkermansia* and *Bifidobacterium* arise, likely influenced by liver disease status, withdrawal, or other clinical factors. Oral investigations also yield inconsistent patterns of *Neisseria*, *Streptococcus*, and other genera.

Cannabis is comparatively understudied, with only six included publications. While some show decreased alpha diversity or distinct community clustering, others detect no meaningful group‐level differences. Taxonomically, cannabis‐related results vary widely. In the oral cavity, certain studies document an expansion of *Streptococcus* or *Actinomyces* (Luo et al. 2021), whereas others note the opposite trend (Newman et al. 2019), highlighting the possibility that site‐specific effects (e.g., saliva vs. oropharynx) may overshadow any consistent cannabis‐related microbiome shift. The limited study number and small sample sizes preclude firm conclusions about cannabis‐associated compositional patterns.

Stimulant research centers predominantly on methamphetamine (Fulcher et al. 2018; Cook et al. 2019; Yang, Yu, Liu, et al. 2021; Deng et al. 2021, 2024, 2022; Wang, Wang, et al. 2023; He et al. 2023; Liu et al. 2023, 2024; Yang, Yu, Yang, et al. 2021; Wang, Feng, et al. 2024), along with a few cocaine studies (Martinez et al. 2022; Fu et al. 2022). In gut‐focused work (Martinez et al. 2022; Fulcher et al. 2018; Cook et al. 2019; Yang, Yu, Liu, et al. 2021; Deng et al. 2021, 2024; Wang, Wang, et al. 2023; He et al. 2023; Liu et al. 2023, 2024), alpha diversity is often reduced (Yang, Yu, Liu, et al. 2021; Wang, Wang, et al. 2023; Liu et al. 2023, 2024), and most investigators observe significant beta‐diversity clustering, implying a relatively uniform microbial response to stimulant use. SCFA‐producing genera such as *Faecalibacterium* and *Bifidobacterium* commonly decline, while opportunistic or proinflammatory taxa (e.g., *Escherichia–Shigella*) often rise. Some data suggest that gender might be a significant confounder, leading to differences in gut microbiome composition (Wang, Wang, et al. 2023; Deng et al. 2022). Oral studies are fewer but typically show decreased alpha diversity and contradictory genus‐level changes, possibly reflecting small cohorts or differences in cocaine versus methamphetamine use. Although these findings suggest a more cohesive pattern associated with stimulant use than with alcohol or cannabis, further work is needed to verify any definitive microbial profile.

Opioid findings frequently involve patients with comorbidities, such as T2D, cirrhosis, or ICU admission, complicating interpretation. Still, several investigations identify increased *Actinobacteria* and *Bifidobacterium*, suggesting a possible impact of opioid therapies—particularly methadone—on these beneficial taxa. Meanwhile, SCFA producers such as *Roseburia* often decline. Certain subpopulations demonstrate unique patterns: individuals on MMT were associated with an increased *Bacteroidetes*/*Firmicutes* ratio but decreased *Akkermansia muciniphila* , a microbe important for gut barrier function (Cruz‐Lebrón et al. 2021). Together, these observations align with associations between opioid treatments and increased abundances of beneficial taxa such as *Bifidobacterium*, but the heterogeneity underscores that neither opioid analgesics nor maintenance treatments are linked to a uniform microbial signature across different clinical contexts.

Despite the diversity of study designs, a few broad observations emerge across all substance categories. First, alpha‐diversity changes were the least consistent for alcohol, while stimulant and opioid investigations tended to show more uniform decreases in alpha diversity. Second, significant beta‐diversity clustering was reported more frequently than alpha‐diversity shifts, suggesting that substance use may induce compositional community changes even when overall diversity remains unchanged. Third, certain taxa (e.g., *Faecalibacterium*, *Roseburia*) were frequently observed at lower abundance in substance users, potentially diminishing gut barrier integrity and anti‐inflammatory capacities. Conversely, opportunistic taxa (e.g., *Escherichia–Shigella* in stimulants, *Proteobacteria* expansions) and pathobionts (e.g., *Streptococcus* in certain contexts) were often reported at higher abundance, although the strength of these signals varied by drug type and population.

On the other hand, although certain genera are frequently labeled “beneficial” or “pathogenic,” interpreting microbiome alterations solely within a “good versus bad” framework oversimplifies the complex host–microbe interactions involved. Substance use likely drives microbiome changes through immune, endocrine, and neural pathways, with diet and nutritional status further influencing these dynamics. For example, increased caloric intake during methadone maintenance therapy may favor expansions of *Bifidobacterium* or *Lactobacillus* (Li et al. 2020) whereas in cirrhotic or diabetic populations, disease‐specific factors might dominate microbial shifts. Additionally, polysubstance use, tobacco smoking, and medication use (e.g., metformin) further confound microbiome associations, underscoring the complexity and need for cautious interpretation.

Beyond bacteria, a small but growing body of work reports associations between substance use and fungal (mycobiome) and viral (virome) community alterations. Alcohol‐related studies, for instance, describe elevated levels of *Candida* and *Saccharomyces* alongside broader fungal community disruptions. Virome analyses similarly show shifts in bacteriophage populations that target genera like *Streptococcus* or *Lactococcus*, with some changes reversing during abstinence. Though preliminary, these findings indicate that substance use is associated with alterations across multiple facets of the gut ecosystem, not just its bacterial component.

To fully understand these microbiome dynamics, it is important to consider the oral–gut axis, which describes the dynamic relationship between oral and intestinal microbiota interacting via enteric (swallowing saliva), hematogenous (oral microbes entering the bloodstream via mucosal breaches), and immune (migration of orally primed T cells) pathways (Xu et al. 2025). Although healthy individuals maintain strong barriers that limit oral‐to‐gut microbial transmission, these defenses can be compromised in disease states such as gastrointestinal inflammation, leading to significant enrichment of oral bacteria in the gut (Dubinkina et al. 2017; Park et al. 2021; Dinakaran et al. 2018). Colonization of the gut by oral pathobionts can disturb the intestinal microbiota and damage the gut barrier, resulting in systemic inflammation and distal spread (Bull‐Otterson et al. 2013; Imai et al. 2021). This oral–gut microbial translocation is particularly relevant in AUD, where the similarity between oral and gut microbiota increases (Ames et al. 2020; Hu et al. 2023), and typical members of oral taxa—including species of genera *Rothia*, *Streptococcus*, *Neisseria*, *Prevotella*, and *Gemella*—are significantly more abundant in the gut microbiota of AUD patients compared with healthy controls (Atarashi et al. 2017). Overrepresentation of oral bacteria in the gut has also been observed in alcoholic liver cirrhosis (Dubinkina et al. 2017).

Dysbiosis‐induced inflammation in the oral cavity may further exacerbate barrier disruption, facilitating the translocation of oral bacteria, their byproducts, and inflammatory mediators into systemic circulation (Sampaio‐Maia et al. 2016). Endotoxemia is frequently observed in AUD (Jokelainen et al. 2001; Malaguarnera et al. 2014). Notably, studies have demonstrated significantly elevated levels of cytokines and chemokines in the saliva and plasma of individuals with AUD compared with healthy controls, with executive dysfunction negatively correlated with increased proinflammatory cytokines and higher salivary concentrations of *Prevotella* (Rodríguez‐Rabassa et al. 2020). In methamphetamine use disorder, oral dysbiosis is marked by increased salivary IL‐1β and IL‐17, driven by an overgrowth of *Neisseria* (Deng et al. 2022).

While most research isolates oral and gut microbiomes, integrative studies in SUDs remain limited. These findings underscore the broader relevance of oral–gut microbial crosstalk in SUDs, as translocation of oral microbiota may exacerbate gut dysbiosis, systemic inflammation, and gut–brain axis dysfunction. Targeting the oral microbiome thus emerges as a promising therapeutic strategy to mitigate these effects in SUD populations, highlighting the need for integrative research to unravel these complex interactions.

Longitudinal research sheds light on whether microbiome changes are transient or more persistent. While short‐term abstinence (e.g., 2–3 weeks) appears insufficient to drive notable microbial recovery (Leclercq et al. 2014; Maccioni et al. 2020; Yang, Yu, Yang, et al. 2021), extended abstinence intervals (e.g., 2 months after methamphetamine cessation) are linked to partial restoration of beneficial taxa and reduced levels of opportunistic species (Liu et al. 2024). Even then, microbiomes of formerly substance‐dependent individuals often remain distinct from those of healthy controls (Piacentino et al. 2024), suggesting that microbiome recovery likely requires sustained substance‐free intervals. This recovery may also be moderated by underlying comorbidities (e.g., liver disease, T2D) and additional interventions (e.g., diet, probiotics).

Nevertheless, many existing studies rely on cross‐sectional designs that limit causal conclusions. Study populations vary significantly; substance use definitions (e.g., heavy use vs. SUD) are inconsistent, and confounders may be underreported. Heterogeneous sequencing techniques further hinder direct comparisons. Consequently, robust longitudinal protocols with standardized sampling methods, repeated measures, and comprehensive dietary data are imperative to determine whether and when the microbiome truly stabilizes, and whether observed microbial shifts are active drivers of substance use or merely byproducts. Ultimately, a clearer understanding of these mechanisms will be crucial for developing and evaluating targeted microbiome interventions (e.g., probiotics, fecal microbiota transplantation, or dietary regimens) to augment standard SUD therapies.

## 5 Conclusions

In summary, research across alcohol, stimulant, opioid, and cannabis studies indicates that psychoactive substance use is associated with measurable alterations in both the gut and oral microbiomes. Beta‐diversity analyses often reveal clear differences between users and controls, while alpha‐diversity findings and specific taxonomic shifts vary among studies. Beneficial short‐chain fattyacid‐producing genera such as *Faecalibacterium* and *Roseburia* are frequently reported at lower abundance, whereas opportunistic or proinflammatory microorganisms tend to expand. Differences in study design, clinical subpopulations, and sample sizes make it challenging to draw definitive conclusions. Findings from abstinence or intervention trials suggest that extended substance‐free intervals may promote partial microbial recovery; however, a full return to a normal profile remains uncertain and may depend on comorbidities or additional treatments. To advance our understanding of how substance use impacts the microbiome, longitudinal, multi‐omics studies with carefully defined clinical phenotypes, standardized protocols, and comprehensive assessments of diet and other confounders are essential. Ultimately, such research may inform microbiome‐based therapies that complement existing SUD treatments.

## Author Contributions

**Artūras Barkus:** conceptualization, methodology, writing – original draft, investigation, formal analysis. **Vaida Baltrūnienė:** conceptualization, methodology, investigation, writing – original draft, formal analysis. **Lina Barkienė:** writing – review and editing, formal analysis. **Justė Baušienė:** writing – review and editing, formal analysis. **Tomas Baltrūnas:** writing – review and editing, formal analysis. **Marius Brazys:** writing – review and editing, formal analysis. **Kornelija Rauduvytė:** writing – review and editing, visualization. **Paulina Kazlauskaitė:** visualization, writing – review and editing. **Augustinas Baušys:** conceptualization, methodology, writing – review and editing, supervision.

## Conflicts of Interest

The authors declare no conflicts of interest.

## Supporting information


**Table S1.** Methodological heterogeneity and confounder assessment across included studies. The table summarizes study characteristics, such as study design, quality assessment using the Newcastle‐Ottawa Scale (NOS) or adapted NOS, and documentation of potential microbiome confounders, including polysubstance use, dietary assessment, antibiotic/probiotic use, comorbidities, and socioeconomic or lifestyle factors.

## Data Availability

All data analyzed in this review were obtained from previously published, publicly available sources (see References). Extracted summary data are provided in the main manuscript tables, and the full risk‐of‐bias and confounder assessment is available in Table S1. No new datasets were generated. Additional information can be obtained from the corresponding author upon reasonable request.
