# Supplementary material for: Exploring the Gut and Oral Microbiomes in Psychoactive Substance Use: A Scoping Review of Clinical Studies
Source: J Neurochem. 2025 Jul 25;169(7):e70165. doi: 10.1111/jnc.70165 (PMC12291152; doi:10.1111/jnc.70165)
Supplement: Supplementary file 1 — Table S1. Methodological heterogeneity and confounder assessment across included studies. The table summarizes study characteristics, such as study design, quality assessment using the Newcastle‐Ottawa Scale (NOS) or adapted NOS, and documentation of potential microbiome confounders, including polysubstance use, dietary assessment, antibiotic/probiotic use, comorbidities, and socioeconomic or lifestyle factors. [file JNC-169-0-s001.docx]

|  | First Author | Publication Year | Study Type | NOS or adapted NOS | Polysubstance use assessed? | Diet assessed? | Antibiotics / probiotics use recorded? | Major comorbidities  reported? | Socioeconomic / lifestyle factors adjusted? |
| --- | --- | --- | --- | --- | --- | --- | --- | --- | --- |
|  | Mutlu EA (38) | 2012 | Case-control | 5 | No | No | Yes (excluded recent antibiotic users) | Yes, partial | No |
|  | Kakiyama G (39) | 2014 | Cross-sectional observational | 4 | No | No | No | No | No |
|  | Leclercq S (40) | 2014 | Prospective  cohort | 7 | No | No | Yes (excluded users ≤2 months) | Yes, partial | No |
|  | Tsuruya A (41) | 2016 | Case-control | 5 | Only tobacco smoking | No | Yes (excluded recent antibiotic users) | Yes, partial | No |
|  | Yang AM (42) | 2017 | Case-control | 4 | No | No | No | No | No |
|  | Dubinkina VB (43) | 2017 | Case-control | 7 | No | No | Yes (excluded users <1 month) | Yes, extensive exclusion | No |
|  | Liao M (44) | 2018 | Cross-sectional observational | 3 | Partial | Yes (21-item FFQ) | Yes (excluded antibiotic/probiotic use ≤6 months) | Yes, partial | No |
|  | Kosnicki KL (45) | 2019 | Cross-sectional observational | 5 | No | Yes (dietary habits collected) | No | No | No |
|  | Lang S (46) | 2020 | Case-control | 5 | No | No | No | No | No |
|  | Zhao W (47) | 2020 | Case-control | 6 | Only tobacco smoking | Yes (all subjects had the "traditional Chinese diet") | Yes (excluded users ≤3 months) | Yes, extensive exclusion | No |
|  | Bjørkhaug ST (48) | 2019 | Case-control | 5 | No | No | Yes (excluded users <1 month) | Yes, extensive exclusion | No |
|  | Duan Y (49) | 2019 | Case-control | 5 | No | No | Yes (excluded antibiotic/immunosuppressive use ≤2 months) | Yes, extensive exclusion | No |
|  | Seo B (50) | 2020 | Cross-sectional observational | 8 | No | No | Yes (excluded users ≤3 months) | Yes, extensive exclusion | No |
|  | Addolorato G (51) | 2020 | Case-control | 7 | No | No | Yes (excluded antibiotic/probiotic drugs ≤4 weeks plus daily laxatives, PPIs, probiotics, pre-/pro-kinetics, β-blockers) | Yes, extensive exclusion | No |
|  | Rodríguez-Rabassa M (52) | 2020 | Case-control | 5 | Yes (smoking, cannabis and other drug habits recorded) | Yes (125-item FFQ) | No | Recorded, not excluded | Minimal |
|  | Smirnova E (53) | 2020 | Case-control | 5 | No | No | Yes (excluded any antibiotic use) | Yes, extensive exclusion | No |
|  | Adams LA (54) | 2020 | Cross-sectional observational | 6 | No | Yes (3-day food record in 87/122 participants) | Yes (excluded current antibiotic/immunosuppressant/PPI use) | Yes, extensive exclusion | No |
|  | Maccioni L (55) | 2020 | Mixed:  Case-control + prospective cohort | 9 | No | No | Yes (excluded antibiotic/probiotic/glucocorticoid/NSAID ≤2 months) | Yes, extensive exclusion | No |
|  | Ames NJ (56) | 2020 | Prospective cohort | 5 | Yes (drug and smoking habits recorded at baseline) | Yes (DHQ-II & meal records) | Yes (excluded antibiotic use ≤1 month; logged if during admission) | Yes, extensive exclusion | No |
|  | Jiang L (57) | 2020 | Case-control | 5 | No | No | Yes (excluded antibiotic/immunosuppressant use ≤2 months) | Yes, extensive exclusion | No |
|  | Maffei VJ (58) | 2021 | Cross-sectional observational | 8 | Yes (tobacco smoking and other drug use captured and modelled) | No | Yes (antibiotic use ≤4 months adjusted as covariate) | Yes, extensive exclusion | No |
|  | Lin R (59) | 2020 | Cross-sectional observational | 7 | Only tobacco smoking | No | Yes (excluded antibiotics/probiotics/PPIs ≤1 month) | Yes, extensive exclusion | No |
|  | González-Zancada N (60) | 2020 | Case-control | 7 | No | Yes (104-item FFQ) | Yes (excluded antibiotics/probiotics/PPIs ≤2 months) | Yes, extensive exclusion | Minimal |
|  | Gurwara S (61) | 2020 | Case-control | 7 | Only tobacco smoking | Yes (HEI from FFQ) | Yes (excluded antibiotics/PPIs ≤7 days pre-scope; none on procedure day) | Yes, extensive exclusion | No |
|  | Vujkovic-Cvijin I (62) | 2020 | bioinformatics/machine‐learning reanalysis | Not applicable | Yes (smoking and other drug use captured and entered in extended models) | Yes (food-frequency variables used for matching & modelling) | Yes (excluded antibiotics ≤6 months) | Yes, extensive exclusion | No |
|  | Kim M (63) | 2020 | Case-control | 5 | Only tobacco smoking | No | Yes (excluded antibiotics ≤4 weeks) | Yes, partial | No |
|  | Wang Y (64) | 2021 | Cross-sectional observational | 6 | No | Yes (8-item FFQ) | Yes (excluded antibiotic use during pregnancy) | Yes, extensive exclusion | No |
|  | Kwan SY (65) | 2022 | Cross-sectional observational | 6 | Only tobacco smoking | Yes (24-h recall) | Yes (excluded antibiotics/probiotics/PPIs ≤30 days) | Yes, extensive exclusion | No |
|  | Hsu CL (66) | 2022 | Mixed:  Case-control + prospective cohort | 8 | No | Yes (standardized hospital diet; no records) | Yes (excluded antibiotics/probiotics/prebiotics ≤2 months) | Yes, extensive exclusion | No |
|  | Hoang T (67) | 2023 | Cross-sectional observational | 4 | Only tobacco smoking | Yes (semi-quantitative FFQ) | No | Recorded, not excluded | No |
|  | Carbia C (68) | 2023 | Cross-sectional observational | 6 | Yes (smoking, cannabis and other drug histories taken; frequent users excluded) | Yes (FFQ covariates) | Yes (excluded antibiotics/probiotics/prebiotics ≤4 weeks) | Yes, extensive exclusion | No |
|  | Szóstak N (69) | 2023 | Cross-sectional observational | 6 | Yes (smoking status and lifestyle variables collected; analysed with environmental fitting) | Yes (comprehensive FFQ) | No | Recorded, not excluded | Yes |
|  | Zhao K (70) | 2023 | Case-control | 6 | Yes (smoking quantified by Fagerström Test for Nicotine Dependence; other substance use disorders excluded) | Yes (in-hospital standardized diet) | Yes (excluded antibiotics/steroids/microbiota-modulating meds ≤2 months) | Yes, extensive exclusion | No |
|  | Kyaw TS (71) | 2023 | Cross-sectional observational | 3 | No | Yes (Harvard FFQ) | Yes (recorded recent antibiotics/pre-/probiotics for QC) | Recorded, not excluded | No |
|  | Philips CA (72) | 2023 | Prospective  cohort | 8 | No | No | No | Yes, partial | No |
|  | Wang C (73) | 2023 | Case-control | 7 | Yes (other drug addictions explicitly excluded) | No | Yes (excluded antibiotics/probiotics/prebiotics ≤3 weeks) | Yes, extensive exclusion | No |
|  | Qiao NN (74) | 2024 | Cross-sectional observational | 6 | No | No | Yes (excluded antibiotics/probiotics/prebiotics ≤3 weeks) | Yes, extensive exclusion | No |
|  | Hoisington AJ (75) | 2024 | Cross-sectional observational | 7 | Yes (substance use disorder, cannabis and cigarette data captured and included as covariates) | No | Yes (in-hospital antibiotic exposure recorded & modelled) | Yes, extensive exclusion | No |
|  | Piacentino D (76) | 2024 | Case-control | 8 | No | Yes (24-h records; not covariate) | Yes (excluded recent antibiotic/probiotic users) | Recorded, not excluded | No |
|  | Wang X (77) | 2024 | Mendelian randomization and bioinformatics | Not applicable | Not applicable | Not applicable | Not applicable | Yes, extensive exclusion | Not applicable |
|  | Li K (78) | 2024 | Case-control | 6 | Only tobacco smoking | No ("similar eating habits" but no dietary data) | Yes (excluded antibiotics ≤1 month) | Yes, extensive exclusion | No |
|  | Börnigen D (79) | 2017 | Case-control | 8 | Yes (lifetime tobacco smoking, alcohol and marijuana use captured) | No | No | No | No |
|  | Fan X (80) | 2018 | Cross-sectional observational | 2 | Yes (lifetime smoking and lifetime marijuana use captured by questionnaire) | No | Yes (excluded antibiotic use ≤2 months) | Yes, extensive exclusion | No |
|  | Ortiz AP (81) | 2022 | Prospective  cohort | 8 | No | No | No | Yes, partial | No |
|  | Ward G (82) | 2023 | Case-control | 4 | No | No | Yes (excluded antibiotics/probiotics ≤1 month) | Yes, extensive exclusion | No |
|  | Yadav S (83) | 2023 | Cross-sectional observational | 1 | Only tobacco smoking | Yes (vegan status only) | No | Yes, extensive exclusion | No |
|  | Maley SJ (84) | 2024 | Cross-sectional observational | 7 | Only tobacco smoking | Yes (122-item FFQ; adjusted) | Yes (current antibiotic use recorded and excluded in sensitivity analysis) | Yes, extensive exclusion | Yes |
|  | Odendaal ML (85) | 2024 | Cross-sectional observational | 6 | Only tobacco smoking | Yes (diet among covariates) | Yes (quantified recent antibiotic use; adjusted in analyses) | Recorded, not excluded | Yes |
|  | Morgan E (86) | 2024 | Cross-sectional observational | 5 | Yes (binary covariate for any other substance included in models) | No | No | Recorded, not excluded | Minimal |
|  | Panee J (87) | 2018 | Case-control | 7 | Yes (partial: urine toxicology ruled out cocaine, amphetamines, barbiturates, benzodiazepines and opiates; alcohol and cigarettes permitted and history taken but not quantified or adjusted) | No | No | Yes, partial | No |
|  | Newman T (88) | 2019 | Case-control | 7 | Yes (concurrent tobacco smoking excluded; no screening for alcohol or other drugs) | No | Yes (excluded antibiotics ≤1 month) | Yes, partial | No |
|  | Luo Z (89) | 2021 | Case-control | 7 | Yes (urine toxicology for prescription and other illicit drugs; alcohol use measured and adjusted) | No | No | Yes, partial | No |
|  | Martinez SS (90) | 2022 | Case-control | 8 | Yes (cocaine use or non-use determined by self-report or urine toxicology) | Yes (24-h recalls & HEI; not covariate) | Yes (excluded antibiotics ≤3 months) | Yes, extensive exclusion | Minimal |
|  | Fu X (91) | 2022 | Case-control | 7 | Yes (urine toxicology screen and chart review confirmed no concomitant illicit drugs; only cocaine users versus never-users) | No | Yes (excluded recent antibiotics/probiotics) | Yes, extensive exclusion | No |
|  | Fulcher JA (92) | 2018 | Cross-sectional observational | 4 | Yes (questionnaire and urine screen captured marijuana, alcohol, tobacco and other substances; modelled as covariates) | No | No | Recorded, not excluded | Yes |
|  | Cook RR (93) | 2019 | Cross-sectional observational | 7 | Yes (marijuana, cocaine, tobacco, binge drinking and other drugs measured and entered into confounder model) | No | Yes (adjusted past-month antibiotic use) | Recorded, not excluded | Yes |
|  | Yang Y (94) | 2021 | Case-control | 7 | No | No | Yes (excluded antibiotics/probiotics/prebiotics ≤3 months) | Yes, partial | No |
|  | Deng D (95) | 2021 | Case-control | 7 | Yes (active screening and exclusion of other illicit drugs within five years; current alcohol and nicotine use quantified and modelled as covariates) | Partial (extreme diets excluded) | Yes (excluded antibiotics/probiotics/corticosteroids/immunomodulators ≤3 months) | Yes, extensive exclusion | Minimal |
|  | Wang Y (96) | 2023 | Case-control | 8 | No | No | Yes (excluded antibiotics/probiotics/prebiotics ≤3 months) | Yes, extensive exclusion | No |
|  | He L (97) | 2023 | Case-control | 8 | Yes (partial: other illicit substance use excluded within previous twelve months; alcohol and nicotine not quantified) | Yes (standardized meals for ≥2 weeks) | Yes (excluded antibiotics ≤3 months) | Yes, extensive exclusion | No |
|  | Liu L (98) | 2023 | Case-control | 7 | Unclear (methamphetamine cohort required repeat positive urine tests; no formal screening for other psychoactive substances; controls required no history of psychoactive substance use) | Partial (extreme patterns excluded) | Yes (excluded antibiotics/probiotics/corticosteroids/immunomodulators ≤1 month) | Yes, extensive exclusion | No |
|  | Liu W (99) | 2024 | Mixed:  Case-control + prospective cohort | 7 | Partial (subjects with mixed use of opioids, cocaine and marijuana excluded; alcohol and nicotine not controlled) | No | Yes (excluded antibiotics/probiotics ≤1 month) | Yes, extensive exclusion | No |
|  | Deng Z (100) | 2024 | Case-control | 8 | Partial (users of heroin excluded; nicotine recorded and analysed; alcohol not controlled) | No (vegetarian exclusion; no data) | Yes (excluded antibiotics/probiotics/immunomodulators/defecation-drugs ≤1 month) | Yes, extensive exclusion | No |
|  | Yang Y (101) | 2021 | Mixed:  Case-control + prospective cohort | 7 | Yes (daily tobacco smoking allowed; alcohol and other addictive drugs ruled out; methamphetamine history recorded) | Partial ("similar diets"; no data) | Yes (excluded antibiotics/probiotics/pre-/post-biotics ≤3 months) | No | Minimal |
|  | Deng Z (102) | 2022 | Case-control | 7 | Yes (partial: limited other illicit drug confounding; healthy controls had no illicit substance use; alcohol and tobacco not reported) | No | Yes (excluded antibiotics/probiotics/corticosteroids/immunomodulators ≤3 months) | Yes, extensive exclusion | No |
|  | Wang D (103) | 2024 | Case-control | 7 | Yes (questionnaire captured smoking, alcohol and other drug use; 2.9 % of methamphetamine group with additional drug exposure; controls had none) | No ("Normal dietary habit”; no data) | Yes (excluded antibiotics ≤4 weeks) | Yes, extensive exclusion | No |
|  | Acharya C (104) | 2017 | Case-control | 9 | Partial (recent alcohol use and antibiotics excluded; other recreational drugs not documented) | Yes (3-day recall in cohort 2) | Yes (excluded antibiotics, SBP prophylaxis, probiotics) | Yes, extensive exclusion | No |
|  | Barengolts E (105) | 2018 | Cross-sectional observational | 5 | No | No | No | Yes, extensive exclusion | No |
|  | Pettigrew MM (106) | 2019 | Cross-sectional observational | 5 | No | No | Yes (detailed antibiotic prescriptions recorded) | Yes, extensive exclusion | Not applicable |
|  | Li Q (107) | 2020 | Case-control | 6 | No | Yes (1-month FFQ; 12 categories) | Yes (excluded antibiotics/antivirals/antifungals/analgesics/anti-inflammatories/probiotics ≤3 months) | Yes, extensive exclusion | Minimal |
|  | Gicquelais RE (108) | 2020 | Cross-sectional observational | 4 | Yes (30-day use of ten illicit or prescription drug classes captured; alcohol use quantified) | Yes (25-item FFQ; fibre calculation) | Yes (self-reported antibiotic use recorded weekly; adjusted in analyses) | Recorded, not excluded | Minimal |
|  | Cruz-Lebrón A (109) | 2021 | Case-control | 8 | No | No | Yes (recorded recent antibiotic exposure) | Recorded, not excluded | No |
|  | Nguyen CL (110) | 2023 | Prospective  cohort | 8 | No | No | Yes (recorded detailed antibiotic use and analysed) | Recorded, not excluded | No |
|  | Xie B (111) | 2024 | Mixed:  Case-control + prospective cohort | 8 | No | No | No | No | No |
|  | Wu Z (112) | 2021 | Cross-sectional observational | 4 | Yes (cigarette and multiple opioid forms collected; alcohol queried; other drugs not) | No | No | Yes, partial | Minimal |

Table S1. Methodological heterogeneity and confounder assessment across included studies. The table summarizes study characteristics such as study design, quality assessment using the Newcastle–Ottawa Scale (NOS) or adapted NOS, and documentation of potential microbiome confounders, including polysubstance use, dietary assessment, antibiotic/probiotic use, comorbidities, and socioeconomic or lifestyle factors. Abbreviations:
NOS: Newcastle–Ottawa Scale (quality assessment tool for observational studies). FFQ: Food Frequency Questionnaire. DHQ: Diet History Questionnaire. HEI: Healthy Eating Index. PPI: Proton Pump Inhibitor. NSAID: Non-Steroidal Anti-Inflammatory Drug. QC: Quality Control. SBP: Spontaneous Bacterial Peritonitis.

Below is the NOS, explicitly tailored to cross-sectional investigations of how substance use (e.g., alcohol, cannabis, opioids, methamphetamine) is associated with gut or oral microbiome alterations. Each item now carries up to the number of “stars” shown in parentheses. For Comparability, we require adjustment for both age and sex (one star each).

**Revised NOS for Cross‐Sectional Studies of Substance Use and Microbiome Changes**

***(Total = 9 stars: 4 for Selection, 2 for Comparability, 3 for Outcome)***

**SELECTION (4 stars total)**

1. **Representativeness of the substance‐user sample**
   • ★ Truly representative of the target population (all eligible or random sampling).
   • ★ Somewhat representative (non‐random sampling).
   • Selected/convenience sample.
   • No description of sampling.
2. **Sample size justification**
   • ★ Justified and ≥ 50 participants.
   • Not justified (< 50 participants).
3. **Participation (non-response) rate**
   • ★ ≥ 80 % of eligible subjects completed all key assessments (validated substance‐use measure + microbiome sampling).
   • 0 points otherwise (response < 80 % or not reported).
4. **Ascertainment of exposure and microbiome**
   • ★ Both of the following are clearly described and validated:
   – Substance-use assessment (e.g. SCID/DSM interview, AUDIT/TLFB, or biomarker assay).
   – Sequencing-based microbiome protocol (e.g. 16S ≥ 10 000 reads/sample or shotgun ≥ 1 million reads, with details on storage, extraction, primers, platform, taxonomic database).
   • 0 points if either exposure or microbiome methods are missing or unvalidated.

**COMPARABILITY (2 stars total)**

1. **Adjustment for age**
   • ★ Analysis explicitly adjusts for age (e.g. covariate or stratified).
   • 0 points otherwise.
2. **Adjustment for sex**
   • ★ Analysis explicitly adjusts for sex (e.g. covariate or sex-specific results).
   • 0 points otherwise.

**OUTCOME (3 stars total)**

1. **Quality of microbiome assessment (up to 2 stars)**
   - ★★ Fully validated sequencing protocol with complete quality control (for example, specified read depth, use of mock control samples, and clear bioinformatic workflow)
   - ★ Validated protocol missing one key element (for example, sequencing depth or quality control details not fully specified)
   - 0 Incomplete or poorly described protocol
2. **Statistical test (1 star)**
   - ★ Appropriate, clearly described microbiome analyses (e.g. α-/β-diversity tests, differential‐abundance with FDR) with effect estimates (CIs and P values)
   - 0 Inappropriate, missing, or insufficiently described statistical methods
